# High-performance photocatalytic nonoxidative conversion of methane to ethane and hydrogen by heteroatoms-engineered TiO_2_

**DOI:** 10.1038/s41467-022-30532-z

**Published:** 2022-05-19

**Authors:** Wenqing Zhang, Cenfeng Fu, Jingxiang Low, Delong Duan, Jun Ma, Wenbin Jiang, Yihong Chen, Hengjie Liu, Zeming Qi, Ran Long, Yingfang Yao, Xiaobao Li, Hui Zhang, Zhi Liu, Jinlong Yang, Zhigang Zou, Yujie Xiong

**Affiliations:** 1 School of Chemistry and Materials Science, and National Synchrotron Radiation Laboratory, University of Science and Technology of China, 230026 Hefei, Anhui China; 2 Institute of Energy, Hefei Comprehensive National Science Center, 350 Shushanhu Rd, 230031 Hefei, Anhui China; 3 Eco-Materials and Renewable Energy Research Center (ERERC), Jiangsu Key Laboratory for Nano Technology, National Laboratory of Solid State Microstructures, School of Physics, Nanjing University, 210093 Nanjing, Jiangsu China; 4 School of Physical Science and Technology, ShanghaiTech University, 201203 Shanghai, China; 5 State Key Laboratory of Functional Materials for Informatics, Shanghai Institute of Microsystem and Information Technology, Chinese Academy of Sciences, 200050 Shanghai, China

**Keywords:** Photocatalysis, Photocatalysis, Catalyst synthesis

## Abstract

Nonoxidative coupling of methane (NOCM) is a highly important process to simultaneously produce multicarbons and hydrogen. Although oxide-based photocatalysis opens opportunities for NOCM at mild condition, it suffers from unsatisfying selectivity and durability, due to overoxidation of CH_4_ with lattice oxygen. Here, we propose a heteroatom engineering strategy for highly active, selective and durable photocatalytic NOCM. Demonstrated by commonly used TiO_2_ photocatalyst, construction of Pd–O_4_ in surface reduces contribution of O sites to valence band, overcoming the limitations. In contrast to state of the art, 94.3% selectivity is achieved for C_2_H_6_ production at 0.91 mmol g^–1^ h^–1^ along with stoichiometric H_2_ production, approaching the level of thermocatalysis at relatively mild condition. As a benchmark, apparent quantum efficiency reaches 3.05% at 350 nm. Further elemental doping can elevate durability over 24 h by stabilizing lattice oxygen. This work provides new insights for high-performance photocatalytic NOCM by atomic engineering.

## Abstract (web summary)

Photocatalytic nonoxidative coupling of methane (NOCM) emerges as an appealing approach for the production of value-added C2 + hydrocarbons and hydrogen. Here the authors propose a heteroatom engineering strategy to prepare Pd single atoms loaded TiO2 for active and selective photocatalytic NOCM.

## Introduction

Methane, the predominant constituent of natural gas, shale gas and combustible ice, has provoked increasing attention as an important feedstock for energy supply and chemical production^1,2^. Conventionally, direct conversion of CH_4_ to value-added multicarbon (C_2+_) compounds is achieved via thermocatalysis through nonoxidative or oxidative routes^3–5^. Nevertheless, undesired side reactions, such as carbon deposition in nonoxidative routes and CH_4_ overoxidation in oxidative routes, inevitably occur in the thermochemical processes, concomitantly with insufficient selectivity and yield of C_2+_ compounds. Meanwhile, high temperatures (700−1110 °C) and pressure (>1 MPa) are required for such thermocatalytic approaches, resulting in high energy consumption and capital input^6,7^. Under such circumstances, future development demands new approaches to direct methane conversion in mild condition.

In this context, photocatalytic nonoxidative coupling of methane (NOCM), which requires only solar energy as energy input and CH_4_ as a reactant, emerges as an appealing approach for the production of value-added C_2+_ hydrocarbons and hydrogen^8,9^. Given strong oxidation capability and lattice oxygen reactivity, metal oxide photocatalysts have been mainly explored for photocatalytic NOCM^10–12^. Despite that, the use of metal oxide unavoidably results in unsatisfying photocatalytic NOCM selectivity and durability. In detail, the valence band of metal oxide is predominantly composed of O 2*p* states^13,14^, enabling the photogenerated holes to concentrate on O sites for cleaving C–H bond of CH_4_. Once C–H bonds are cleaved, overoxidation of CH_4_ would readily take place at the O sites while consuming lattice oxygen^15,16^. As an outcome, photocatalytic reactivity decays by losing lattice oxygen while overoxidation products, e.g., CO_2_ and CO arise^17,18^, restricting the product selectivity and catalyst durability of the oxide-based photocatalytic NOCM.

To overcome these limitations, the valence band of oxide photocatalysts should be rationally engineered. In the past decade, metallic single atoms (SAs) loaded nanostructures have attracted tremendous interest for photocatalysis^19^, one of whose advantages is their capability of tailoring photocatalyst electronic structure^20^. The incorporation of metallic *d*-orbital structure shows more occupied state in valence band maximum (VBM) and provides additional active sites in photocatalysts^21,22^. Against this background, we propose that tailoring VBM of oxide photocatalyst with metallic SAs could be a feasible approach to reduce the contribution of O 2*p* states to valence band near-surface, suppressing the overoxidation of CH_4_ with lattice oxygen.

Here, taking TiO_2_, a widely used oxide photocatalyst as a proof-of-concept model, we report that incorporation of Pd SAs in oxide surface lattice can overcome the limitations upon screening a series of metallic SAs toward photocatalytic NOCM. According to the simulation of the material, the Pd–O_4_ unit demonstrates the largest contribution to VBM of surface TiO_2_ so as to accumulate photogenerated holes, which in turn dissociate C–H bonds at Pd SAs. Furthermore, the unique feature of Pd in stabilizing ∙CH_3_ allows it to be the reaction site for coupling of reaction intermediates. As a result, the Pd SAs modified catalyst (Pd_1_/TiO_2_) exhibits the highest performance toward C_2_H_6_ production, with an apparent quantum efficiency of 3.05% at 350 nm and an extraordinary C_2_H_6_ production rate of 0.91 mmol g^–1^ h^–1^. More importantly, the occupied state of Pd–O_4_ in VBM suppresses the overoxidation of CH_4_ with lattice oxygen, which offers 94.3% selectivity toward C_2_H_6_ production and substantially improved stability of oxide photocatalyst. Furthermore, a pertinent element (i.e., Si) is selected to dope the TiO_2_ lattice for further stabilizing subsurface lattice oxygen to elevate catalyst durability over 24 h.

## Results

### Material screening and photocatalytic performance

**Fig. 1:**
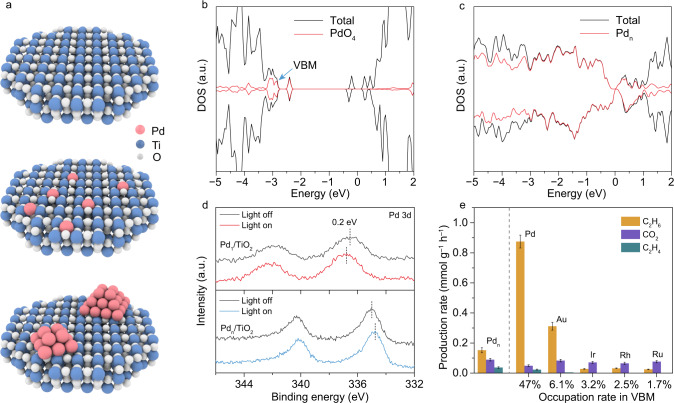
Screening of metallic SAs with large contributions to VBM of M_1_/TiO_2_ for photocatalytic NOCM. **a** Schematic illustration for TiO_2_ (001)-based models—pristine TiO_2_ (top), Pd_1_/TiO_2_ (middle), and Pd_n_/TiO_2_ (bottom). **b**, **c** Total density of states (TDOS) of Pd_1_/TiO_2_ (**b**) and Pd_n_/TiO_2_ (**c**). The red line represents the contribution of metals to TDOS, in which Pd–O_4_ unit show the highest proportion in VBM (highlighted by blue arrow). **d** Pd 3d XPS spectra of Pd_1_/TiO_2_ and Pd_n_/TiO_2_ under light irradiation indicating charge accumulation. **e** The relationship of metal contribution in VBM and photocatalytic NOCM performance for various M_1_/TiO_2_ as well as Pd_n_/TiO_2_. Reaction condition: 0.1 MPa CH_4_, 3 h light irradiation. The error bars represent the standard deviation of the experiments.

To screen metallic SAs with the largest contribution to VBM, we employ density functional theory (DFT) calculation to examine various SAs modified TiO_2_ (M_1_/TiO_2_) catalysts near the surface. The key to the reconstruction of VBM is the coordination of SAs with lattice oxygen. To this end, we select TiO_2_ (001) plane as the surface for decorating metallic SAs because it contains relatively loosely packed oxygen atoms as illustrated in Fig. 1a, which allows the formation of the M–O bond between the metallic SAs and lattice oxygen of TiO_2_^23^. For metallic nanoparticles, such a bond structure can hardly be formed so that they are mainly loaded on the TiO_2_ surface (Pd_n_/TiO_2_). Figure 1b, c shows the simulated density of states (DOS) for various M_1_/TiO_2_ catalysts (see Supplementary Fig. 1 for other calculation results). For M_1_/TiO_2_ catalysts, the composition of VBM is significantly altered although the decorated metallic SAs induce impurity states in the bandgap of TiO_2_. Pd–O_4_ unit exhibits the largest contribution to VBM among all the studied catalysts, implying the potential of accumulating photogenerated holes. Notably, for the Pd nanoparticles, the photogenerated electrons would be concentrated at Pd due to the formation of Schottky junction (Fig. 1c)^19,24^, while the photogenerated holes are remained in TiO_2_. Such an assumption is verified by Pd 3d X-ray photoelectron spectroscopy (XPS, Fig. 1d). Specifically, under light irradiation, the binding energy for Pd_1_/TiO_2_ is positively shifted for 0.2 eV, indicating the accumulation of photogenerated holes at Pd–O_4_ unit. In contrast, Pd_n_/TiO_2_ displays negatively shifted binding energy in Pd 3d XPS upon light irradiation (Supplementary Fig. 2). The charge accumulation is also proven by infrared spectroscopy with CO probe (Supplementary Fig. 3). Based on the theoretical simulation results, we prepare a series of metal SAs (Pd, Au, Rh, Ir, and Ru) modified TiO_2_ for photocatalytic NOCM as shown in Fig. 1e. Generally, the TiO_2_ loaded with Pd SAs, which owns the largest contribution to VBM of TiO_2_, demonstrates the highest activity and selectivity toward CH_4_−to−C_2_H_6_ conversion. Moreover, computational and experimental results show that TiO_2_ (001) is the optimal surface for loading Pd SAs as compared with other TiO_2_ facets (Supplementary Fig. 4).

**Fig. 2:**
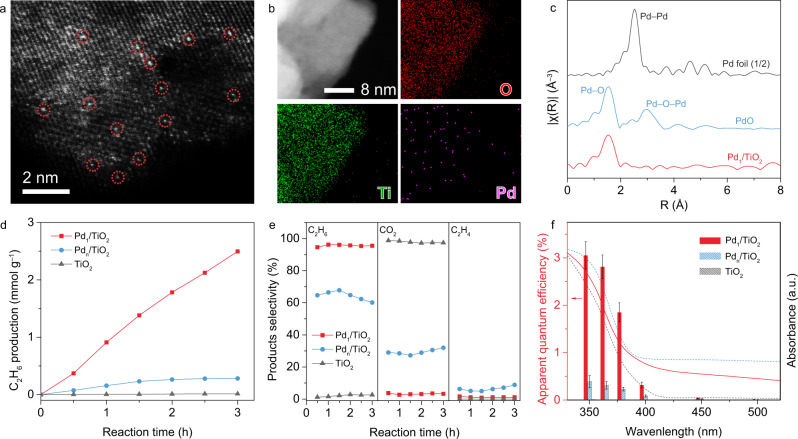
Structural characterization and photocatalytic NOCM performance of Pd_1_/TiO_2_ catalyst. **a**–**c** STEM image (**a**), elemental mappings (**b**) and Pd K-edge EXAFS spectra in *R* space (**c**) of Pd_1_/TiO_2_. The isolated Pd atoms are marked with red circles in (**a**). **d**, **e** The C_2_H_6_ production rates (**d**) and the corresponding selectivity of C_2_H_6_, C_2_H_4_, and CO_2_ (**e**) in 0.1 MPa CH_4_. **f** The AQEs for CH_4_−to−C_2_H_6_ conversion with their UV-vis diffuse reflectance spectra as references. The error bars represent the standard deviation of the experiments.

In this context, we focus on the investigation of the photocatalytic NOCM process on Pd_1_/TiO_2_ in reference to pristine TiO_2_ and Pd_n_/TiO_2_ with similar Pd content (Supplementary Fig. 5 and Supplementary Table 1). Typically, all the samples are based on TiO_2_ nanosheets dominated with (001) surface (Supplementary Figs. 6 and 7). To resolve the structure of Pd SAs, the Pd_1_/TiO_2_ catalyst is examined by a series of microscopic and spectroscopic techniques. The high-angle annular dark-field scanning transmission electron microscopy (HAADF-STEM) images of Pd_1_/TiO_2_ indicate that the Pd elements are atomically dispersed on the support (Fig. 2a and Supplementary Fig. 8), which is also verified by energy-dispersive X-ray spectroscopy (EDS) mapping (Fig. 2b). To investigate the coordination environment of Pd in Pd_1_/TiO_2_ catalyst, the catalyst is further characterized by synchrotron radiation-based X-ray absorption near-edge structure (XANES) spectroscopy (Supplementary Fig. 9). As revealed by extended X-ray adsorption fine structure (EXAFS) spectra in *R* space for Pd K-edge (Fig. 2c), only a single peak for Pd–O coordination (1.53 Å) can be observed on Pd_1_/TiO_2_, confirming the domination of isolated Pd atoms in Pd_1_/TiO_2_ catalyst. According to the fitting, the average coordination number is determined to be 4 (Supplementary Table 2)^25^. The presence of isolated Pd atoms on the TiO_2_ can be further affirmed via investigating the CO adsorption behavior on the catalysts (Supplementary Fig. 2)^26,27^. We thus conclude that the Pd atoms in Pd_1_/TiO_2_ are isolated from each other and coordinated with four O atoms in TiO_2_ to form Pd−O_4_ units.

Upon acquiring structural information for the catalyst, the detailed photocatalytic NOCM measurements are performed at room temperature under 600 mW cm^–2^ xenon arc lamp irradiation. As shown in Fig. 2d, pristine TiO_2_ nanosheets exhibit a negligible C_2_H_6_ production rate. This performance is due to the large contribution of O atoms to VBM, which leads to the direct participation of the lattice oxygen in CH_4_ conversion. In detail, photogenerated holes in TiO_2_ are predominantly concentrated at O atoms to cleave C−H bonds of CH_4_. As the activated methyl species can hardly desorb from O sites, they are vulnerable to overoxidation. As a result, CO_2_ emerges as the major product with nearly 100% selectivity as shown in Fig. 2e. Since Pd nanoparticles play the role of electron accumulation, Pd_n_/TiO_2_ does not suppress overoxidation to CO_2_ but promotes C_2_H_6_ production owing to dehydrogenation and stabilization of ∙CH_3_ at Pd nanoparticles^28,29^. As such, Pd_n_/TiO_2_ exhibits an initial rate of ~0.18 mmol g^–1^ h^–1^ for C_2_H_6_ production in the first 1 h, which decreases gradually after 2 h of light irradiation due to lattice oxygen consumption by overoxidation (Fig. 2d). The overoxidation is evidenced by a large amount of produced CO_2_ with a selectivity of about 32% (Fig. 2e). In stark contrast, Pd_1_/TiO_2_ displays a very high C_2_H_6_ production rate of ~0.91 mmol g^–1^ h^–1^ at the initial 1 h of light irradiation (Fig. 2d). More importantly, catalyst stability has been dramatically improved (Supplementary Fig. 10), yielding C_2_H_6_ at 2.6 mmol g^–1^ with over 93% carbon balance (Supplementary Table 3). The high C_2_H_6_ production rate over Pd_1_/TiO_2_ can be maintained with a 33-fold scale-up of catalyst usage under appropriate reaction conditions (Supplementary Fig. 11). These results clearly demonstrate that the modification of TiO_2_ VBM with Pd SAs can allow the CH_4_ conversion to proceed on the Pd instead of the lattice oxygen of TiO_2_, substantially suppressing overoxidation of CH_4_ with lattice oxygen. As a direct outcome, the C_2_H_6_ selectivity for Pd_1_/TiO_2_ is maintained at ~94.3% (Fig. 2e). Notably, we observe that the measured amount of produced H_2_ is approximately in theoretically stoichiometric ratio with the production of C_2_H_6_ and trace CO_2_ (Supplementary Fig. 12), further confirming the superiority of Pd SAs in enhancing photocatalytic NOCM performance of TiO_2_. As a result, turnover number (TON) reaches as high as 186 toward C_2_H_6_ evolution over the Pd_1_/TiO_2_ catalyst for 3 h light irradiation. In terms of light utilization, the Pd_1_/TiO_2_ catalyst achieves an AQE of 3.05% under 350-nm light irradiation (Fig. 2f). Furthermore, the photocatalytic performance is directly proportional to the light intensity, demonstrating that the activation of CH_4_ is driven by photogenerated holes under mild condition (Supplementary Fig. 13). The outstanding NOCM performance indicates that Pd SAs modification enables an efficient C–C coupling process and suppresses CH_4_ overoxidation. To trace the origin of carbonaceous products, we further perform a series of control and isotope-labeling experiments (Supplementary Figs. 14–16), showing that the CH_4_ is indeed the source of the produced C_2_H_6_.

### Insight into photocatalytic NOCM mechanism

The information gleaned above has recognized the superior performance of CH_4_ conversion by modifying VBM of TiO_2_ with Pd SAs. We are now in a position to look into the reaction mechanism over TiO_2_ with a focus on the role of Pd SAs. Based on photoelectrochemical measurements (Supplementary Fig. 17), it is demonstrated that the presence of Pd can greatly enhance the photogenerated charge carrier separation efficiency of the TiO_2_, and the Pd_1_/TiO_2_ demonstrates the highest efficiency due to the homogeneous dispersion of Pd SAs on the TiO_2_. To gain insight into the intermediates of photocatalytic NOCM on Pd_1_/TiO_2_, electron paramagnetic resonance (EPR) measurement is performed to investigate possible radicals by using 5,5′-dimethyl-1-pyrroline N-oxide (DMPO) as a radical scavenger in a liquid-phase reaction condition^30^. As shown in Supplementary Fig. 18, both the characteristic signals of ∙CH_3_ and ∙OH can be detected using Pd_1_/TiO_2_. Given that the ∙OH is formed due to the presence of water, this observation indicates that the surface ∙CH_3_ is the key intermediate for CH_4_ coupling^31,32^. In comparison, only ∙OH is detected for pristine TiO_2_ and Pd_n_/TiO_2_, suggesting the high efficiency of CH_4_ dissociation on Pd_1_/TiO_2_.

**Fig. 3:**
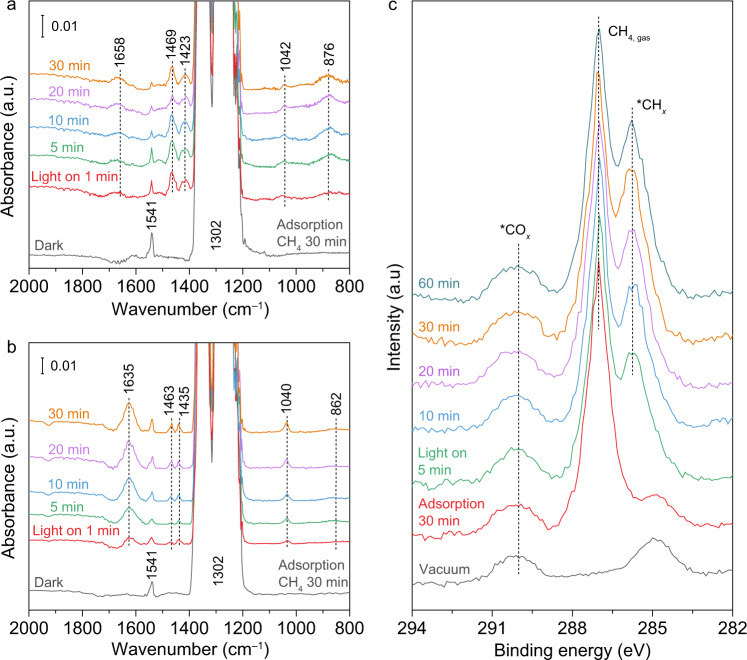
Intermediates detection for photocatalytic NOCM. **a**, **b** In situ DRIFTS spectra for photocatalytic NOCM over Pd_1_/TiO_2_ (**a**) and Pd_n_/TiO_2_ (**b**). **c** In situ high-resolution C 1 s XPS spectra of Pd_1_/TiO_2_ catalyst with different light illumination time in 45 Pa CH_4_.

Furthermore, in situ diffuse reflectance-infrared Fourier-transform spectroscopy (DRIFTS) is employed to investigate the intermediates for photocatalytic NOCM. The catalysts are exposed to CH_4_ under dark condition firstly for adsorption. As shown in Supplementary Fig. 19, apart from the peak at 1302 cm^–1^ corresponding to C–H deformation vibration of CH_4_, the peak at 1541 cm^–1^ can ascribed to the symmetric deformation vibrational mode of CH_4_ on metal oxide surface^17,33^. Upon light irradiation, the vibrational modes of CH_2_/CH_3_ deformation at 1469 and 1423 cm^–1^ appear immediately (Fig. 3a), indicating that CH_4_ is rapidly dissociated over Pd_1_/TiO_2_ surface by photogenerated charge carriers^8^. Furthermore, the peak of C–C stretching (876 cm^–1^) appears and gradually increase with the prolonged light irradiation, demonstrating C–C coupling on catalyst surface^8,34^. Meanwhile, the slight growth of C=O (1671 cm^–1^) and C–O (1042 cm^–1^) stretching vibrational modes are assigned to the species for CO_2_ formation, suggesting the sluggish overoxidation of CH_4_ on Pd_1_/TiO_2_^35–37^. In contrast, intense C=O and C–O stretching vibration signals can be observed for Pd_n_/TiO_2_ (Fig. 3b), while the CH_2_/CH_3_ intermediates can hardly be resolved in the DRIFTS spectra for pristine TiO_2_ (Supplementary Fig. 20). Taken together, photocatalytic NOCM results and in situ DRIFTS characterizations clearly reveal that Pd SAs modification can promote the coupling route and diminish the overoxidation of CH_4_ under light irradiation. The surface hydrocarbon species are also monitored by in situ near ambient pressure XPS (NAP-XPS) studies. After introducing 45 Pa CH_4_ into NAP-XPS chamber under the dark, the peak of gas-phase CH_4_ (287.0 eV) is observed^38^. Subsequently, the peak of surface ∙CH_x_ species at 285.6 eV appears and is gradually boosted along with light irradiation (Fig. 3c), further corroborating the dissociation of CH_4_ on catalyst surface^39,40^. Notably, the fitting curves of the C 1 s spectrum show the absence of C–O formation on the surface (Supplementary Fig. 21). This suggests that no C–O specie is involved in the CH_4_−to−C_2_H_6_ conversion.

**Fig. 4:**
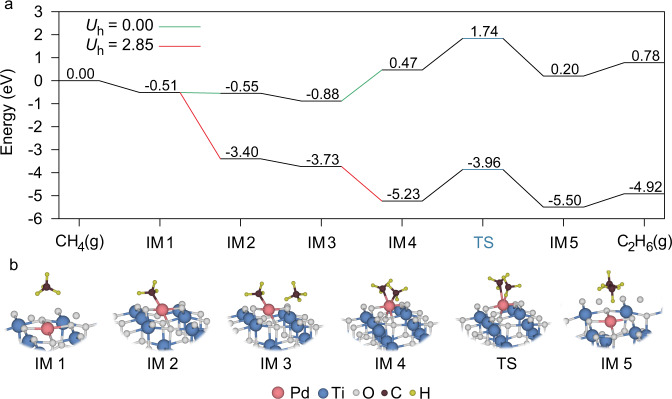
DFT calculations for photocatalytic NOCM. **a** Free-energy diagrams for C_2_H_6_ production on Pd_1_/TiO_2_ with hole energy (*U*_h_) of 0 eV and 2.85 eV. **b** Atomic configurations for the corresponding steps in the simulation.

To have a full image of the coupling process of NOCM, we perform DFT calculation to examine CH_4_ dissociation and intermediate coupling over Pd_1_/TiO_2_ with the assistance of photogenerated holes. In our calculation, Pd atom is stabilized in the fourfold hollow sites of TiO_2_ (001) face to form Pd–O_4_ complex. As demonstrated in our material screening, such a metal−support interaction modulates the VBM of TiO_2_. We, therefore, calculate the potential energy diagram of CH_4_ coupling reaction on the Pd_1_/TiO_2_ surface (Fig. 4a), based on the atomic configurations in Fig. 4b. When CH_4_ is adsorbed on Pd atom, the C–H bond is dissociated by the oxidation of photogenerated holes on Pd–O_4_ unit, releasing H atom and leaving Pd–CH_3_ (IM2) on the surface. Given the steric effect for adsorption, the activation of the second CH_4_ requires a substantially higher energy of 1.35 eV (i.e., green line between IM3 and IM4). As a result, the extremely high activation energy of 1.74 eV (167.9 kJ mol^–1^) from initial state to TS state hinders the NOCM process under dark condition. However, taking the extra potential provided by the photogenerated holes into account (Supplementary Fig. 22), the energy barrier of the step turns into downhill (i.e., red line between IM3 to IM4), followed by the C–C coupling over Pd atom (TS to IM5) and C_2_H_6_ desorption. Meanwhile, we also experimentally measure the activation energy of the NOCM process under light irradiation condition. A relatively low value of 9.54 kJ mol^–1^ is acquired (Supplementary Fig. 23). The dramatic decrease of activation energy under light irradiation suggests that the photogenerated carriers play a crucial role in overcoming the energy barrier of NOCM. Nevertheless, the simulation suggests that the efficiency of C–C coupling should be associated with the probability of the second CH_4_ reaching the active site, depending on the concentration of gaseous CH_4_. In our photocatalytic tests, we indeed recognize that the yield of C_2_H_6_ decreases when reducing the concentration of filling CH_4_ (Supplementary Fig. 24).

### Performance enhancement with lattice oxygen stabilization

**Fig. 5:**
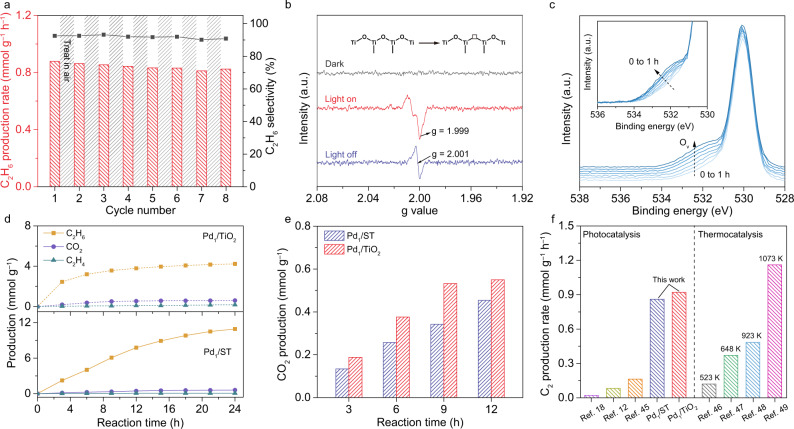
Evidence and prevention strategy for lattice oxygen consumption. **a** Production rates and selectivity of C_2_H_6_ in the cyclic tests by Pd_1_/TiO_2_. Each cycle lasts 3 h, between which the catalyst is treated in air. **b** In situ EPR signals for Pd_1_/TiO_2_ catalyst in 0.1 MPa CH_4_ under different conditions. The inset is the formation of O vacancy during the reaction. **c** In situ high-resolution O 1 s XPS spectra for Pd_1_/TiO_2_ catalyst with different light illumination time in 45 Pa CH_4_. **d**, **e** The comparison of C_2_H_6_ (**d**) and CO_2_ (**e**) production between Pd_1_/TiO_2_ and Pd_1_/ST. **f** The photocatalytic NOCM by Pd_1_/ST and Pd_1_/TiO_2_ benchmarked against the photocatalytic NOCM and the mild-condition thermocatalytic NOCM in literature. The temperatures in thermocatalysis represent the reaction temperatures applied in those works.

As revealed by the photocatalytic NOCM, the C_2_H_6_ is the primary product along with the minor CO_2_ using Pd_1_/TiO_2_. Note that no gaseous oxygen or other oxidizing agent is present in the reactor, implying that the overoxidation of CH_4_ to CO_2_ is derived from lattice oxygen of the catalyst^15–18^. Such oxidation by lattice oxygen is inevitably accompanied with its consumption that will eventually reduce photocatalytic performance. To this end, we investigate the time-dependent NOCM on Pd_1_/TiO_2_ (Supplementary Figs. 25 and 26), showing that the performance of the Pd_1_/TiO_2_ decays after 6 h of reaction and can be recovered by heating the catalyst in air. As such, the photocatalytic performance of Pd_1_/TiO_2_ is well maintained for eight cyclic tests with each cycle of 3 h followed by a recovering process (Fig. 5a). Furthermore, the characterizations including aberration-corrected HAADF-STEM, CO adsorption DRIFTS and EXAFS for Pd_1_/TiO_2_ after the cyclic tests show the chemical state of Pd is well preserved with single dispersion (Supplementary Figs. 27−29). Moreover, the H_2_–temperature-programmed reduction (H_2_–TPR) analysis shows that the Pd–O species in Pd_1_/TiO_2_ are more stable than Pd oxide, indicating the stability of Pd–O_4_ units (Supplementary Fig. 30). Apparently, the overoxidation of CH_4_ to CO_2_ can be suppressed by Pd_1_/TiO_2_ through the contribution of Pd SAs to VBM but still occurs via the consumption of lattice oxygen. On the basis of DFT calculation (Fig. 4b, IM2), we can elucidate the mechanism for the consumption of lattice oxygen on Pd_1_/TiO_2_ that one of Pd–O bonds in Pd–O_4_ unit is fractured to steady the state of [Pd…∙CH_3_] after CH_4_ activation on the Pd atom. As such, the exposed O atom from the fractured Pd–O, together with other O atoms uncoordinated with Pd atoms, can be consumed to produce CO_2_ via overoxidation, resulting in performance decay.

To further prove the mechanism, we employ in situ EPR to examine the structural changes of the catalysts during photocatalytic NOCM. Upon light irradiation, Pd_1_/TiO_2_ shows the signal for −O^•–^ radical sites in anatase crystals at g = 1.999, which is derived from the separation of photogenerated charge carriers and can participate in CH_4_ oxidation (Fig. 5b)^41^. After turning light off, the signal for the single electron trapped O defects at g = 2.001 can be observed, manifesting the emergence of O vacancies after the photocatalytic process^42^. A similar observation is also acquired for pristine TiO_2_ (Supplementary Fig. 31). Furthermore, the appearance of the O vacancy during the reaction can be further attested via the in situ NAP-XPS, corresponding with the results of in situ EPR (Fig. 5c, Supplementary Figs. 32 and 33)^23,43^. As determined by temperature-programmed oxidation (TPO), 0.7 wt.% of lattice oxygen has been consumed in the photocatalytic process (Supplementary Fig. 34). Moreover, the location of the formed O vacancies is determined by ^17^O magic angle spinning nuclear magnetic resonance (^17^O MAS NMR) and EPR spectra. It turns out that the consumption of lattice oxygen preferentially takes place at the O atoms near the Ti sites with 2-fold oxide coordination (OTi_2_) on the catalyst surface (Supplementary Fig. 35).

Given the experimental results indicating the CH_4_ overoxidation process, we further conduct DFT calculation to simulate the possible steps of CH_4_–to–CO_2_ conversion with lattice oxygen consumption. As shown in Supplementary Fig. 36, taking the O site adjacent to Ti atom as an example, the photogenerated holes are required to provide extra potential for CH_4_ dehydrogenation at the O site; otherwise, the high energy barrier of further steps will inhibit the process thermodynamically. Specifically, the activation of the last hydrogen atom in CH_4_ requires a high energy of 1.82 eV (i.e., green line between O-IM4 and O-IM5), which is the rate-determining step for overoxidation process under dark condition. However, the energy barrier of this step turns into downhill with the assistance of photogenerated holes (i.e., red line between O-IM4 and O-IM5), and the bonding between the adsorbed CH_4_ and the second lattice oxygen becomes the rate-determining step with the energy barrier of 1.12 eV (i.e., O-IM3 to O-IM4). This indicates that the stabilization of the lattice oxygen can weaken the bonding between CH_4_ and O site and suppress the overoxidation of CH_4_ to CO_2_ under light irradiation.

The mechanistic study suggests that the stabilization of lattice oxygen should be an efficient method for further suppressing the CH_4_ overoxidation and enhancing the stability of Pd_1_/TiO_2_. To this end, we employ the elemental doping via Si to further stabilize O atoms of TiO_2_ (Supplementary Fig. 37)^44^. As shown in Fig. 5d as well as Supplementary Figs. 38 and 39, incorporating Si into Pd_1_/TiO_2_ (Pd_1_/ST) can mitigate CH_4_ overoxidation to a large extent and maintain photocatalytic activity for up to 24 h. The effect of Si doping on suppressing CH_4_ overoxidation is further verified by the comparison of CO_2_ production between Pd_1_/TiO_2_ and Pd_1_/ST (Fig. 5e). Moreover, the computational results demonstrate that the incorporation of Si can further increase the energy barrier of the rate-determining step in the conversion process of CH_4_ to CO_2_ as compared with that of Pd_1_/TiO_2_, indicating that the presence of Si atoms in TiO_2_ can suppress the overoxidation of CH_4_ by stabilizing the lattice oxygen atoms (Supplementary Fig. 40). It is worthwhile to note that the photocatalytic performance of optimized Pd_1_/TiO_2_ and Pd_1_/ST well exceed the existing photocatalysts for NOCM and reach the level of thermocatalysts working between 923 and 1073 K (Fig. 5f and Supplementary Table 4)^45–49^.

## Discussion

To address the limitations in photocatalytic NOCM, we have developed a photocatalyst based on TiO_2_ by modifying valence band near-surface with Pd**–**O_4_ units, which achieves highly efficient light-driven CH_4_ conversion to C_2_H_6_ under mild condition. Benefiting from the contribution of Pd**–**O_4_ units to valence band, the single Pd sites display the accumulation of photogenerated holes, which are able to dissociate C–H bond and adsorb ∙CH_3_ for C_2_H_6_ formation. In the meantime, reducing the role of O atoms in valence band substantially suppresses the overoxidation of CH_4_ with lattice oxygen, dramatically improving catalytic durability. As a result, the catalyst exhibits the ultrahigh C_2_H_6_ production rate of 0.91 mmol g^–1^ h^–1^ and selectivity of 94.3%, reaching the level of thermocatalytic NOCM at relatively mild condition. Fundamentally, the in situ generation and retention of O vacancies on photocatalysts during photocatalytic NOCM have been experimentally observed and assigned to the consumption of lattice oxygen for CO_2_ generation, providing key information for further performance enhancement. Interestingly, the overoxidation process can be suppressed by steadying subsurface lattice oxygen with lattice doping, further enhancing catalyst reusability. This work highlights the importance of rational lattice engineering to tuning reaction pathways, and is expected to offer guideline for the design of high-performance catalysts toward NOCM at mild condition.

## Methods

### Catalysts preparation

TiO_2_ nanosheets were prepared by a simple hydrothermal method. Typically, 10 mL of tetrabutyl titanate (TBOT) was mixed with 1.6 mL HF with stirring for 30 min. Then the solution was transferred into a 50 mL Teflon-lined autoclave and reaction at 180 °C for 24 h. After that, the resulting white slurry was centrifuged and washed with distilled water several times and dried in a vacuum oven. For the synthesis of Pd_1_/TiO_2_ catalyst, 20 mg TiO_2_ nanosheets were dispersed in 5 mL dilute ammonia solution (pH = 10.5) to form a homogeneous white suspension. Separately, 20 μL Pd(NO_3_)_2_ solution (21.1 mM) was attenuated with dilute ammonia solution (pH =  10.5) to 3 mL. Next, the 3 mL mixture was injected into the TiO_2_ suspension with stirring by using the syringe pump at a rate of 1 mL h^–1^, followed by vigorous stirring for another 3.5 h. The obtained slurry was then washed with distilled water until the pH turns neutral and dried in the oven at 60 °C. After that, the collected solid was calcined in air at 300 °C in air for 2 h. The Pd_n_/TiO_2_ was prepared by reduction of NaBH_4_. Generally, 60 mg of TiO_2_ nanosheets were dispersed in 30 mL distilled water, then 60 μL Pd(NO_3_)_2_ solution (21.1 mM) was added into the mixture and further react with 5 mg NaBH_4_. The product was separated by centrifugation, washed with water several times and dried in a vacuum oven at 60 °C overnight. The other metal SAs loaded M_1_/TiO_2_ catalysts were synthesized with the similar electrostatic adsorption wet impregnation technique by adding different metallic precursor solution with approximate content.

For Si-doping TiO_2_, 150 μL Tetraethoxysilane, 10 mL TBOT, and 1.2 mL HF were added into anhydrous ethanol (20 mL) with stirring for 30 min. The precursor solution was transferred into 50 mL Teflon-lined autoclave and reaction at 180 °C for 2 h. The Pd_1_/ST was obtained by a similar procedure with Pd_1_/TiO_2_.

### Characterization

Powder X-ray diffraction patterns were recorded by using a Philips X’Pert Pro Super X-ray diffractometer with Cu–Kα radiation (*λ* = 1.54178 Å). Transmission electron microscopy (TEM) images were taken on a Hitachi Model H-7700 microscope at 100 kV. High-resolution TEM (HRTEM) images were taken on a JEOL JEM-2100 field-emission higher-resolution transmission electron microscope at 200 kV. The aberration-corrected HAADF-STEM images and corresponding EDS mapping profiles were measured on the JEOL ARM-200F field-emission transmission electron microscope operated at 200 kV. UV-Vis diffuse reflectance data were recorded in the spectral region of 200–800 nm with a Shimadzu SolidSpec-3700 spectrophotometer. The X-ray adsorption fine structure (XAFS) spectra of sample were acquired at beamline 1W1B of the Beijing Synchrotron Radiation Facility (BSRF) and beamline 14W1 of the Shanghai Synchrotron Radiation Facility (SSRF). For XANES spectra, the experimental absorption coefficients as a function of energies were processed by background subtraction and normalization procedures and reported as the normalized intensity. The acquired EXAFS data were processed according to the standard procedure using the WinXAS3.1 program. The in situ NAP-XPS measurements were carried at the beamline BL02B1 of SSRF under light irradiation (365 nm LED light) or dark condition. In situ EPR spectra for oxygen vacancies detection are obtained on the JEOL JES-FA200 spectrometer equipped with a 500 W xenon lamp as the illumination source.

### Detection of methyl radical (∙CH_3_)

5, 5'-dimethyl-1-pyrroline N-oxide (DMPO) was used to trap the generated methyl or hydroxyl radicals in the reaction system. Briefly, the as-prepared catalysts and DMPO are dispersed in ice-bath DI water. The mixture was vigorously shaken for 10 s and irradiated under 0.1 MPa CH_4_ by using a 500 W xenon lamp, and then analyzed by EPR spectroscopy.

### DRIFTS for CO adsorption

DRIFTS measurements were performed using a Bruker IFS 66 v Fourier-transform spectrometer equipped with Harrick diffuse reflectance accessory with ZnSe and quartz window at BL01B in the National Synchrotron Radiation Laboratory (NSRL) in Hefei, China. After the sample was loaded into the reaction chamber, pure Ar (99.999%) was purged into the chamber firstly for background spectra collection. Subsequently, CO was introduced into the system until CO adsorbed saturation. Then the pure Ar was purged to remove the gaseous CO and the spectra were collected by averaging 128 scans at a resolution of 2 cm^–1^.

### In situ DRIFTS for CH_4_ activation

In situ DRIFTS measurements were performed using a Bruker IFS 66 v Fourier-transform spectrometer equipped with Harrick diffuse reflectance accessory with ZnSe and quartz window at BL01B in the NSRL in Hefei, China. Each spectrum is recorded by averaging 128 scans at a resolution of 2 cm^–1^. After sample loading, the pure Ar was purged into the chamber for background spectra collection. Subsequently, pure CH_4_ (99.999%) is introduced into the chamber for 10 min. For CH_4_-adsorption process, the spectra were collected under dark condition at 5, 10, 20, and 30 min, respectively. After that, the system was exposed to light irradiation and the spectra were collected when the irradiation times are 1, 5, 10, 20, and 30 min, respectively.

### Photocatalytic CH_4_ conversion measurements

The photocatalytic CH_4_ conversion experiments were carried out in CH_4_ atmosphere (0.1 MPa) at room temperature in a 30 mL custom-made quartz tube reactor. In a typical test, 3.0 mg of catalyst is dispersed in 800 μL of DI water and then dropped onto a 1 × 3 cm clear glass and dried in air at 323 K. After the glass was vertically placed in the reactor, 1 vol %, 10 vol % CH_4_/Ar or pure CH_4_ was purged into the system. The reactor was irradiated by a 300 W xenon lamp (PLS-SXE300, Perfect light). The produced H_2_ and hydrocarbons were quantified by a gas chromatograph (GC, 7890B, Ar carrier, Agilent) equipped with thermal conductivity detector (TCD) and flame ionization detector (FID). Another GC (Techcomp GC-7900, China) equipped with a TDX-01 packed column was employed to determine the amounts of CO and CO_2_. The isotope-labeled experiments were performed by using pure ^13^CH_4_, ^12^CH_4_, or CD_4_ as feeding gas. The products were analyzed with gas chromatography-mass spectrometry (GC–MS, 7890 A and 5975 C, He carrier, Agilent). For the regeneration treatment, the catalyst was heated under 80 °C for 2 h in air.

### Determination of AQE for C_2_H_6_ production

The AQE was obtained by using different monochromatic filters (350, 380, 400, 450, 500, and 600 nm) during light irradiation. The irradiance values in ultraviolet region were measured by an UV energy meter (UV DESIGN, UV-int 140). The AQE was calculated through the following equation:

1 $${AQE}=\frac{{N}_{e}}{{N}_{p}}=\frac{n\times M\times {N}_{A}}{\frac{W\times S\times t}{h\times v}}\times 100 \%$$

where *N*_*e*_ and *N*_*p*_ represent the number of reacted electrons and incident photons, respectively, n is the number of electrons transferred during C_2_H_6_ formation, M is the molar number of C_2_H_6_, *N*_*A*_ is the Avogadro’s constant, and *W*, *S*, *t*, *h*, and *v* are the light intensity, irradiation area, irradiation time, Planck constant and light frequency, respectively.

### Photocurrent measurements

The photoelectrochemical measurements were performed on CHI 660E electrochemical workstation (CH Instruments) in three-electrode system under light irradiation. In all, 3.0 mg of catalyst was dispersed in 800 μL of DI water and then dropped onto a 1 × 3 cm fluorine-doped tin oxide (FTO)-coated glass for employed as work electrode. The Pt foil and saturated Ag/AgCl electrode were used as counter electrode and reference electrode, respectively. 0.5 M Na_2_SO_4_ aqueous solution was chosen as the electrolyte. The electrolyte was saturated with pure Ar or CH_4_ prior before the tests. The photocurrent responses of the prepared photoelectrodes (i.e., I–t) were operated by measuring the photocurrent densities under chopped light irradiation (light on/off cycles: 50 s) at a bias potential of 0.8 V vs. Ag/AgCl for 800 s.

### Calculation of carbon balance

Carbon balance was calculated through the following equation:

2 $${{{{{\rm{Carbon\; balance}}}}}}=\frac{{Moles}\,{of}\,{products}\,{in}\,{terms}\,{of}\,{carbon}}{{Moles}\,{of}\,{methane}\,{consumed}}\times 100 \%$$

### Computational method

The spin-polarized density functional theory (DFT) calculations were performed by using the Vienna ab initio simulation package (VASP)^50,51^. The interaction between the core and valence electrons was described using the frozen-core projector augmented wave (PAW) approach^52,53^. The generalized gradient approximation of Perdew–Burke–Ernzerhof (PBE) functional was used^54^. The energy cutoff was set to be 400 eV. The DFT-D2 vdW correction proposed by Grimme was adopted to describe long-range vdW interactions^55^. The convergence criteria of energy and force were set to be 10^−5^ eV and 0.02 eV Å^−1^, respectively. A Monkhorst–Pack grid of 9 × 9 × 3 k-points was used to sample the Brillouin zone of the unit cell for the anatase TiO_2_. The optimized lattice parameters were a = b = 3.789 Å and c = 9.479 Å. A 3 × 3 supercell with five layers of TiO_2_ was used to describe the (001) surface. The bottom layer Ti and O dangling bonds were saturated with pseudo-hydrogens with nuclear charges of +1.25 and +0.75, which has been reported in previous studies^56,57^. A vacuum space greater than 15 Å perpendicular to the surface was applied to separate the interactions between neighboring slabs. The Brillouin zone was sampled by the 1 × 1 × 1 *Γ*-centered k-point for the slab system.

The energy differences (Δ*E*) for the reactions were calculated according to the approach originally proposed by Nørskov and coworkers^58,59^, Δ*E* = Δ*E*^0^ + Δ*E*_U_, where Δ*E*^0^ is the computed reaction energy. The external potential supplied by photogenerated holes was added by Δ*E*_U_ = −e*U*_h_, where *U*_h_ is the electrode potential relative to the standard hydrogen electrode (SHE). The transition states were searched with the nudged elastic band method with climbing images^60^.

## Supplementary information

Supplementary Information

## Data Availability

All data supporting the findings of this study are available in the article and its Supplementary Information.  Source data are provided with this paper.

## Source data

Source Data
