# Supplementary material for: High-performance photocatalytic nonoxidative conversion of methane to ethane and hydrogen by heteroatoms-engineered TiO2
Source: Nat Commun. 2022 May 19;13:2806. doi: 10.1038/s41467-022-30532-z (PMC9119979; doi:10.1038/s41467-022-30532-z)
Supplement: Supplementary file 1 — Supplementary Information [file 41467_2022_30532_MOESM1_ESM.pdf]

Supplementary Information

High-performance photocatalytic nonoxidative conversion of methane to ethane and hydrogen by heteroatoms-engineered TiO_2

Wenqing Zhang[1,2,¶], Cenfeng Fu[1,¶], Jingxiang Low[1,¶], Delong Duan[1], Jun Ma[1], Wenbin Jiang[1], Yihong Chen[1], Hengjie Liu[1], Zeming Qi[1], Ran Long[1]*, Yingfang Yao[3]*, Xiaobao Li[4,5], Hui Zhang[4,5], Zhi Liu[4,5], Jinlong Yang[1], Zhigang Zou[3] and Yujie Xiong[1,2]*

[1] School of Chemistry and Materials Science and National Synchrotron Radiation Laboratory, University of Science and Technology of China, Hefei, Anhui 230026, China.

[2]Institute of Energy, Hefei Comprehensive National Science Center, 350 Shushanhu Rd. Hefei, Anhui 230031, China

[3]Eco-Materials and Renewable Energy Research Center (ERERC), Jiangsu Key Laboratory for Nano Technology, National Laboratory of Solid State Microstructures, School of Physics, Nanjing University, Nanjing, Jiangsu 210093, China

[4]School of Physical Science and Technology, ShanghaiTech University, Shanghai 201203, China.

[5]State Key Laboratory of Functional Materials for Informatics, Shanghai Institute of Microsystem and Information Technology, Chinese Academy of Sciences, Shanghai 200050, China.

[¶]These authors contributed equally

*Corresponding author.
Tel: 0086-0551-63606657, E-mail: longran@mail.ustc.edu.cn; yaoyingfang@nju.edu.cn; yjxiong@ustc.edu.cn

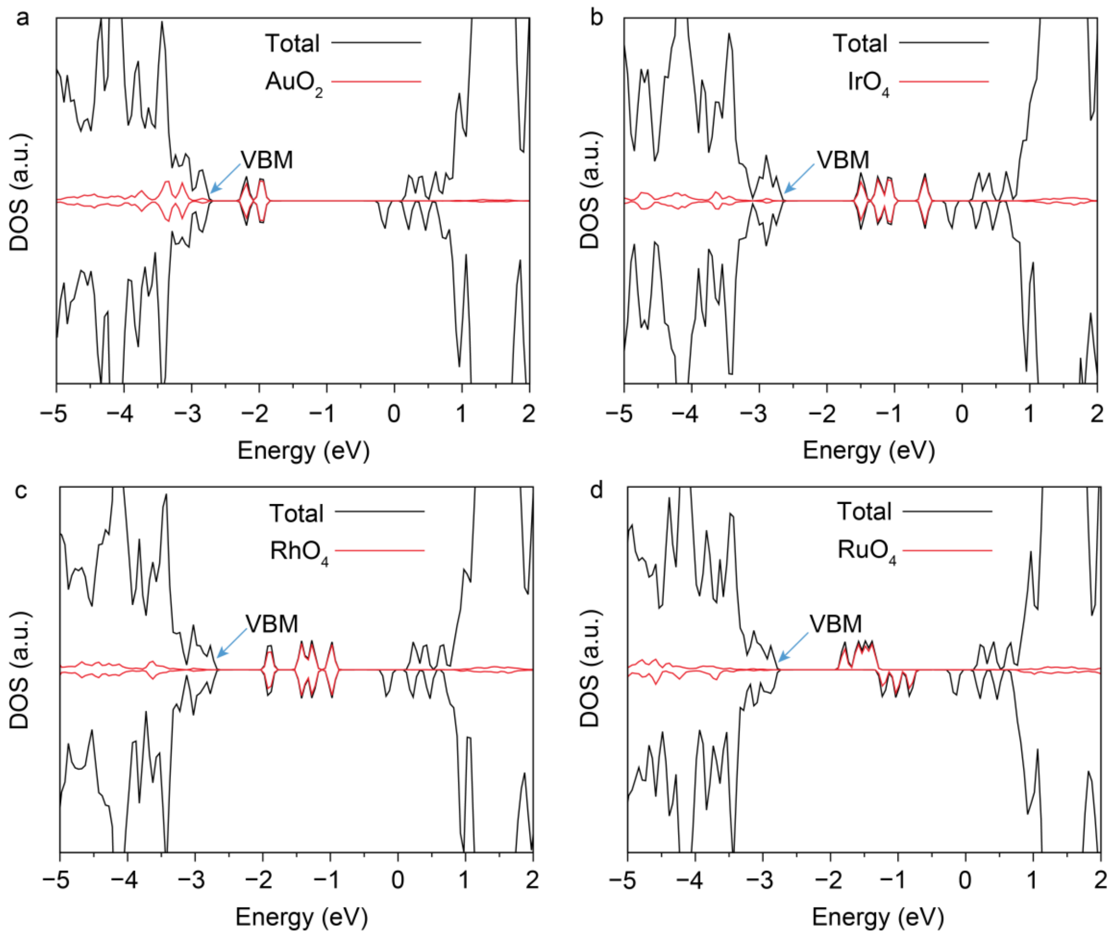

Supplementary Fig. 1 Total density of states (TDOS) of M_1/TiO_2. (a) Au_1/TiO_2, (b) Ir_1/TiO_2, (c) Rh_1/TiO_2 and (d) Ru_1/TiO_2. The red line represents the contribution of metallic SAs to TDOS.

The Au atom shows 6.1% occupied state in valence band maximum (VBM) (Supplementary Fig. 1a), which also increases the NOCM activity on Au_1/TiO_2. However, Ir, Rh and Ru atoms only show 3.2%, 2.5% and 1.7% occupied states in VBM of their M_1/TiO_2 structures, respectively (Supplementary Fig. 1b–d), indicating that the photoinduced holes will be accumulated at O sites rather than in M–O_x unit, leading to the inert performance for C_2H_6 production.

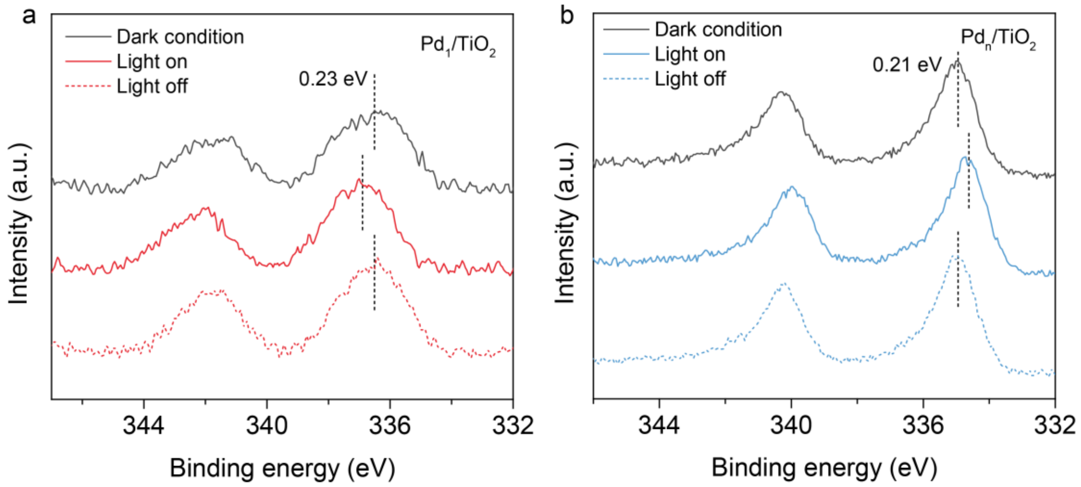

Supplementary Fig. 2 Pd 3d XPS spectra of Pd/TiO_2 with light switched on/off. (a) Pd_1/TiO_2 and (b) Pd_n/TiO_2.

Under light irradiation, the binding energy of Pd 3d XPS for Pd_1/TiO_2 is positively shifted and that of Pd_n/TiO_2 is negatively shifted. When the light is switched off, both the shifts of binding energy for Pd_1/TiO_2 and Pd_n/TiO_2 return to the original states under dark condition.

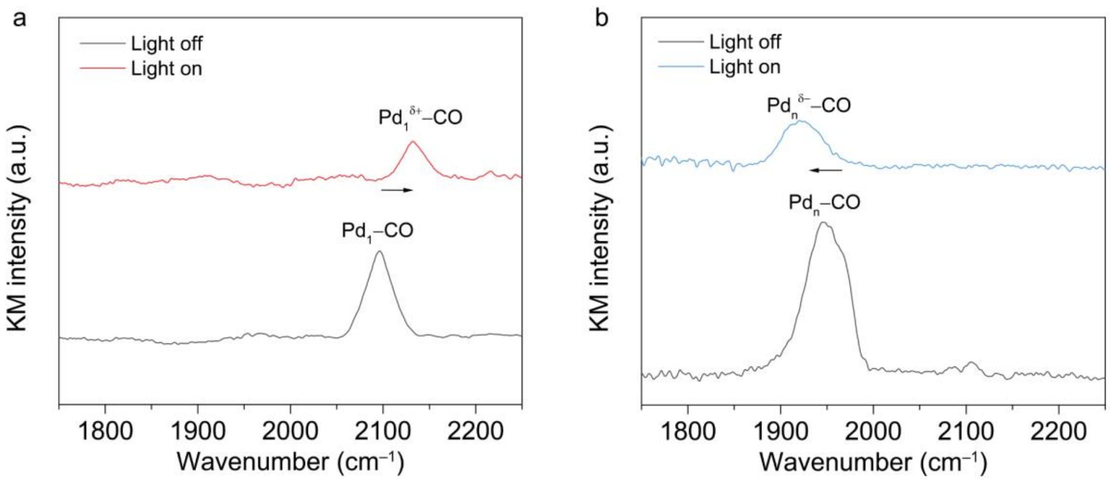

Supplementary Fig. 3 CO adsorption DRIFTS spectra of Pd/TiO_2 with/without light irradiation. (a) Pd_1/TiO_2 and (b) Pd_n/TiO_2.

During light irradiation, the photogenerated hole of Pd_1/TiO_2 will be accumulated in Pd–O_4 unit, resulting in the more positively charged Pd_1 site. As a probe molecule, the CO chemisorbed to the Pd site will lose electrons, which thus increases the vibrational frequency of CO in diffuse reflectance-infrared Fourier transform spectroscopy (DRIFTS) (Supplementary Fig. 3a). In contrast, the photogenerated electron of Pd_n/TiO_2 will be transferred to Pd nanoparticle under light irradiation, leading to the more negatively charged Pd sites[1]. Such a feature can be resolved by the reduction of CO vibrational frequency in DRIFTS (Supplementary Fig. 3b). The CO adsorption DRIFTS also provides a tool for examining the dispersion state of Pd sites. Unlike the broad and large signals of CO adsorbed on bridge or hollow sites on Pd nanoparticles, only one peak around 2100 cm^{-1} attributed to the linear CO adsorption configuration is observed, which further verifies the form of isolated Pd atoms[2].

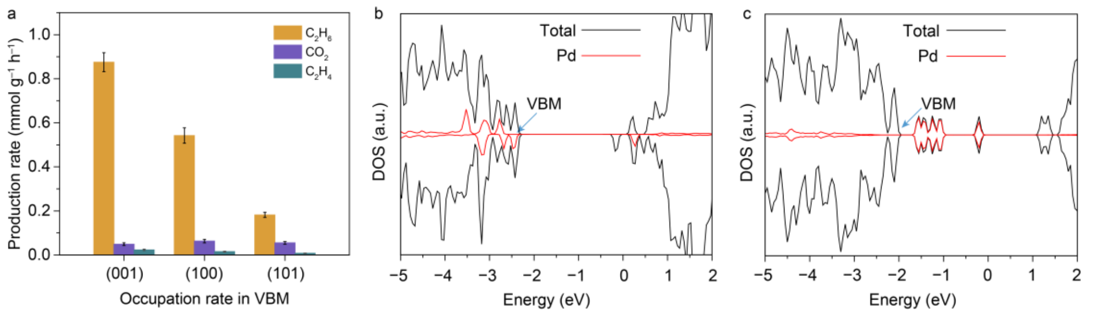

Supplementary Fig. 4 Performance and calculation for Pd_1/TiO_2 with different TiO_2 facets. (a) The photocatalytic NOCM performance for Pd_1/TiO_2 with different TiO_2 facets. Reaction condition: 0.1 MPa CH_4, 3 h light irradiation. Total density of states (TDOS) of Pd_1/TiO_2 on TiO_2 (b) (100) and (c) (101) facets, respectively. The error bars represent the standard deviation of the experiments.

We prepare the TiO_2 nanocrystals dominated with (100) and (101) planes according to the previous report and further loaded them with Pd SAs respectively for photocatalytic NOCM measurements (Supplementary Fig. 4a)[3]. The catalytic results show that TiO_2 (001) facet with Pd SAs loading exhibits the highest activity and selectivity of C_2H_6 production. To further validate the correlation between the lattice plane and its electron structure of the catalyst, we calculate the density of states (DOS) of Pd_1/TiO_2 with (100) or (101) facet of the supports (Supplementary Fig. 4b and 4c). The Pd single atoms only show 21% and 0.8% occupied states in VBM of their Pd_1/TiO_2 (100) and Pd_1/TiO_2 (101) structures, respectively, lower than the 47% occupied states in VBM of Pd_1/TiO_2 (001). The computational results indicate that the photogenerated holes prefer to be accumulated at O sites in the cases of (100) and (101) facets, limiting the coupling of methane at Pd sites. Overall, the TiO_2 (001) loaded with Pd SAs, which owns the largest contribution to VBM of Pd_1-TiO_2, exhibits the highest activity and selectivity toward CH_4–to–C_2H_6 conversion.

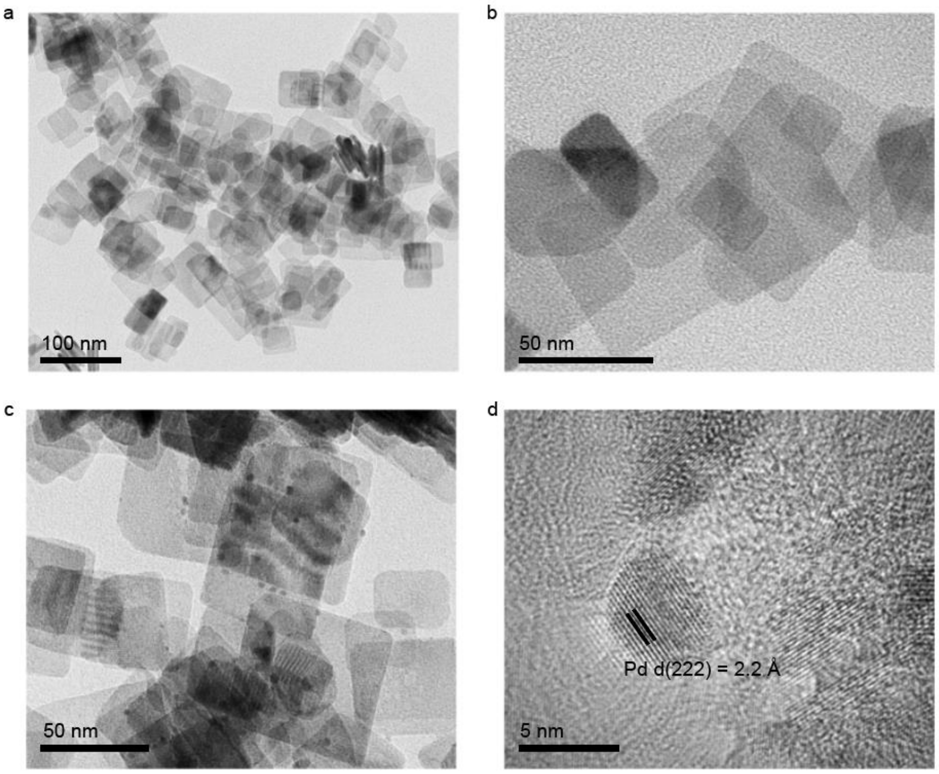

Supplementary Fig. 5 Electron microscopy characterization of TiO_2 nanosheets and Pd_n/TiO_2. (a, b) TEM images of TiO_2 nanosheets. (c) TEM and (c) HRTEM images of Pd_n/TiO_2.

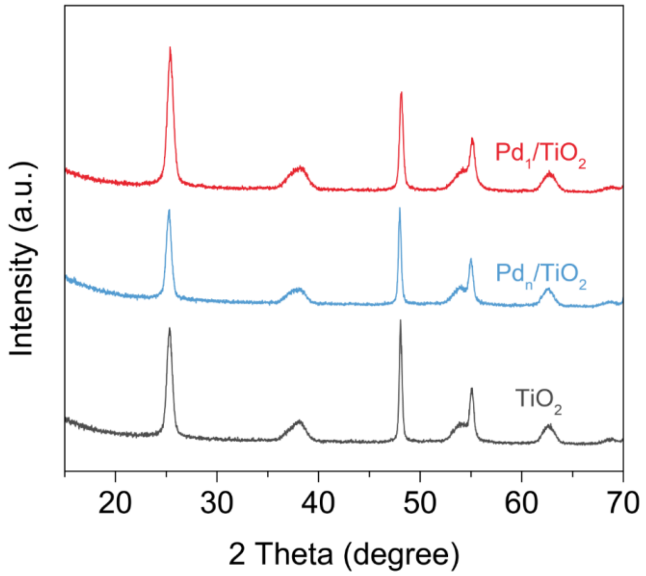

Supplementary Fig. 6 XRD characterization of the samples. XRD patterns of TiO_2, Pd_1/TiO_2 and Pd_n/TiO_2 samples.

In X-ray diffraction (XRD) pattern of Pd_1/TiO_2, only the peaks of anatase TiO_2 are observed (Supplementary Fig. 6), suggesting that Pd atoms are highly dispersed on TiO_2 nanosheets.

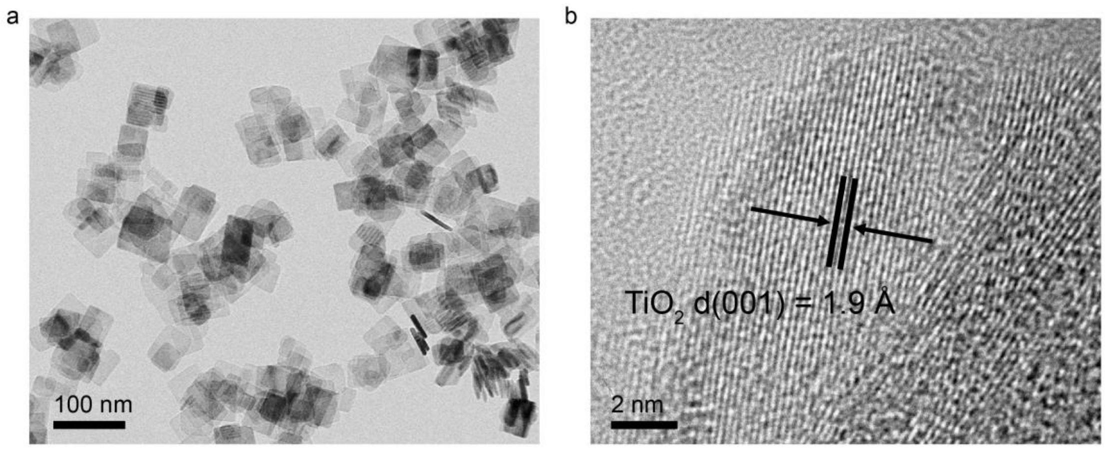

Supplementary Fig. 7 Electron microscopy characterization of Pd_1/TiO_2 catalyst. (a) TEM and (b) HRTEM images of Pd_1/TiO_2 catalyst.

Transmission electron microscopy (TEM) reveals that TiO_2 is of well-defined nanosheet structure (Supplementary Fig. 7a). As confirmed by high-resolution TEM (HRTEM, Fig. 7b), the nanosheets are bounded with (001) surface and no Pd nanoparticles are observed on the nanosheets.

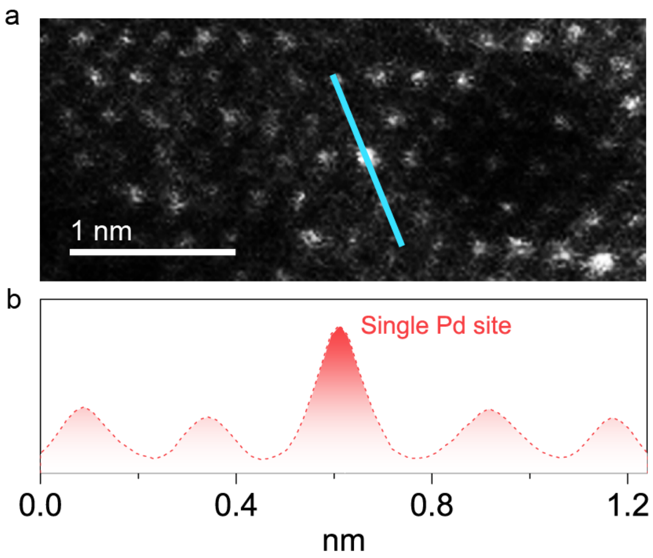

Supplementary Fig. 8 Atomic-resolution electron microscopy characterization of Pd_1/TiO_2 catalyst. (a) Aberration-corrected STEM image and (b) the corresponding intensity profile recorded for Pd_1/TiO_2 catalyst.

Moreover, the aberration-corrected scanning transmission electron microscopy (STEM) image and exemplary line profile in Supplementary Fig. 8 further demonstrate that the Pd atoms are presented as single atoms.

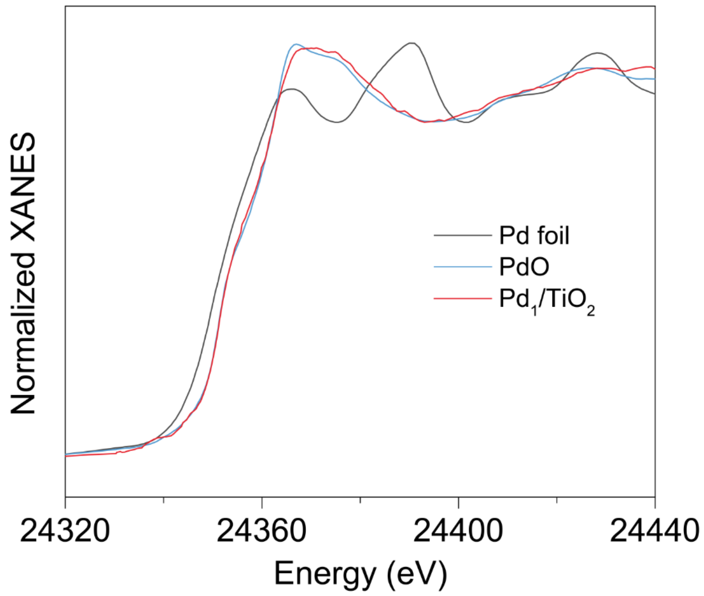

Supplementary Fig. 9 XANES characterization of Pd_1/TiO_2. Normalized Pd K-edge X-ray absorption near-edge structure (XANES) spectra of Pd foil, PdO and Pd_1/TiO_2.

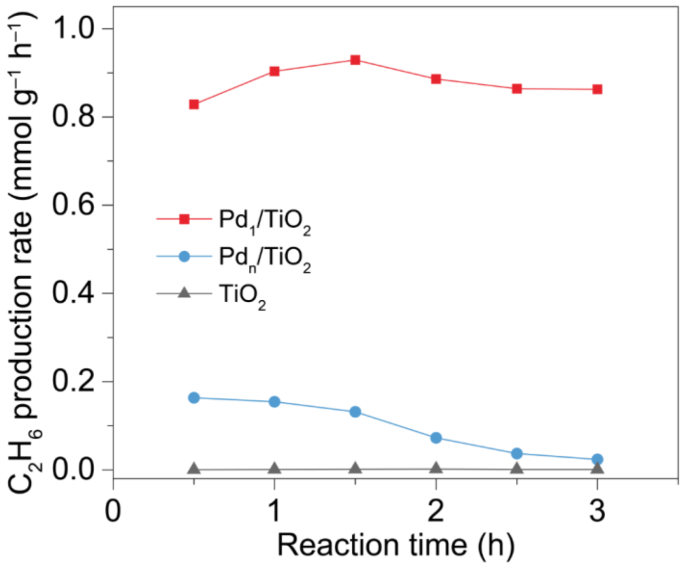

Supplementary Fig. 10 NOCM performance of the samples. C_2H_6 production in photocatalytic nonoxidative coupling of methane (NOCM) over TiO_2, Pd_1/TiO_2 and Pd_n/TiO_2 catalysts in 0.1 MPa CH_4.

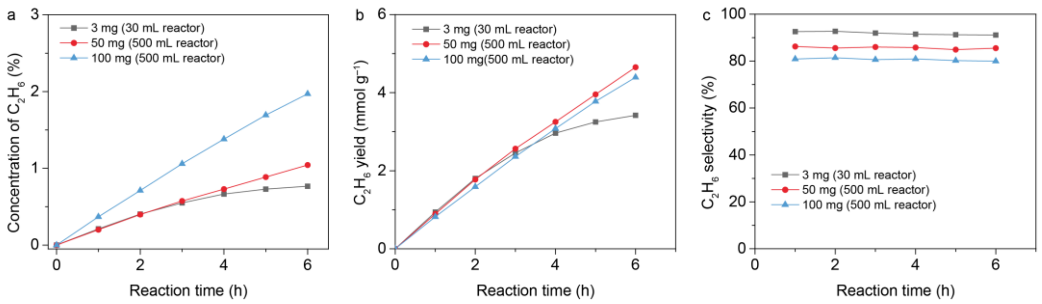

Supplementary Fig. 11 NOCM performance of Pd_1/TiO_2 with difference catalyst dosage. (a) The C_2H_6 yield concentration, (b) C_2H_6 production rates and (c) C_2H_6 selectivity of Pd_1/TiO_2 with difference catalyst dosage.

We employ the 500 mL quartz reactor with catalyst coating at the bottom. The concentration of C_2H_6 can reach 2.3% after 6 h light irradiation when using 100 mg catalyst, and the C_2H_6 yields on 50 and 100 mg catalysts are both higher than 0.8 mmol g^{-1} h^{-1}, indicating that the excellent NOCM performance can maintain at the usual amounts of catalysts with appropriate reaction condition. Moreover, raising the amount of catalyst can improve the catalytic stability for more than 6 h by increasing the amount of surface lattice oxygen on catalyst. The selectivity for C_2H_6 is slightly reduced when more catalyst is used, which may be ascribed to the contact between the methane and the surface lattice oxygen without Pd coordination. Overall, a 33-fold scale-up of the catalytic system shows the maintained catalytic activity and brings only ~10% reduction in selectivity, which presents the robustness of this catalytic system.

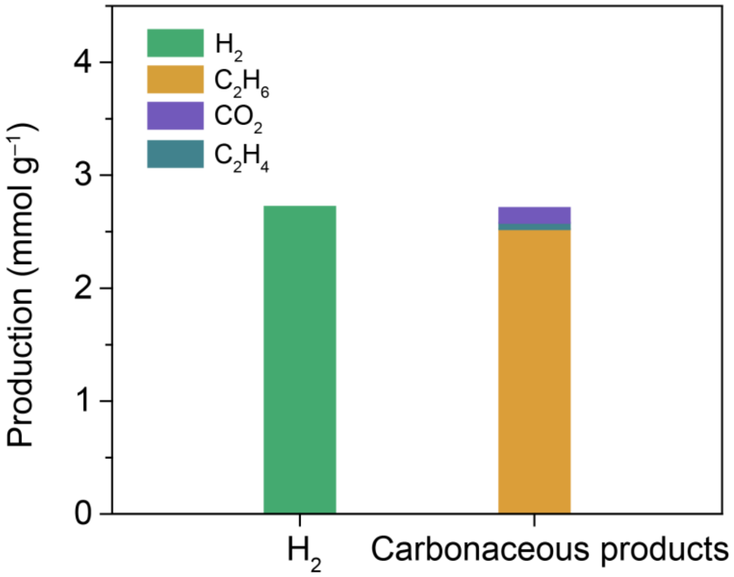

Supplementary Fig. 12 The products from NOCM. The production of carbonaceous products and H_2 in photocatalytic NOCM over Pd_1/TiO_2 after 3 h light irradiation.

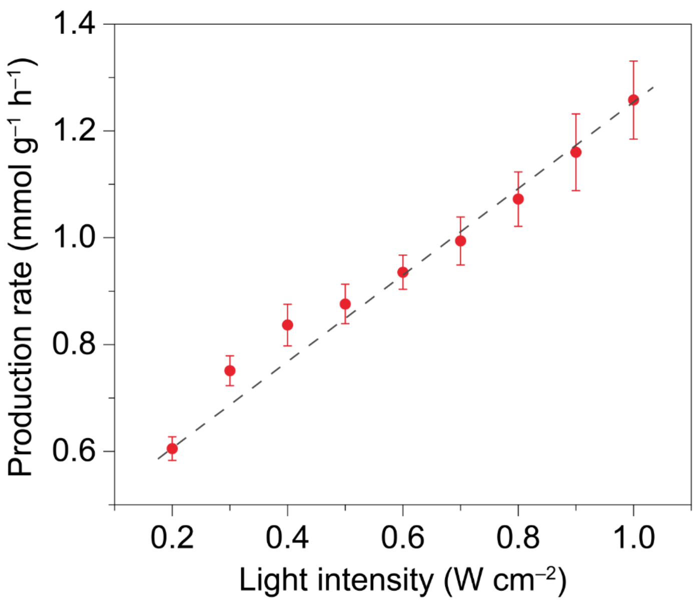

Supplementary Fig. 13 NOCM performance under different light intensity. Production rates of carbonaceous products in photocatalytic NOCM over in the first 3 h under different light intensity. The error bars represent the standard deviation of the experiments.

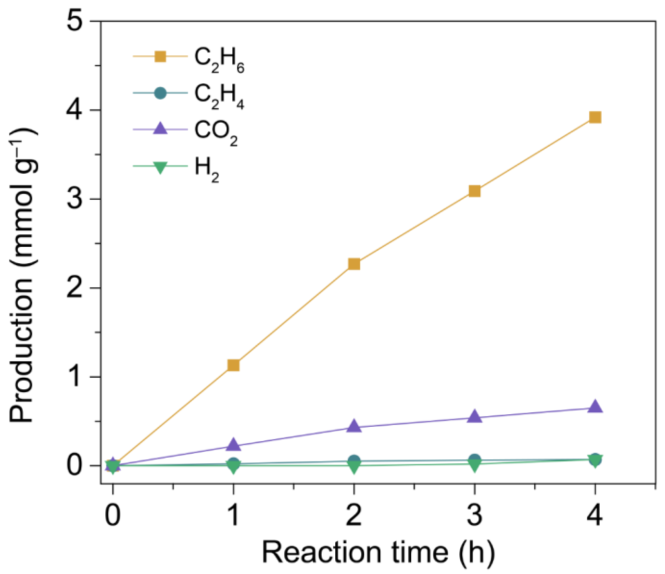

Supplementary Fig. 14 Time-dependent NOCM performance. Time-dependent production of carbonaceous products and H_2 in photocatalytic methane conversion over Pd_1/TiO_2 in the presence of trace O_2. Reaction condition: 3 mg of photocatalytic, 22.3 μmol O_2 in pure CH_4, 0.1 MPa pressure at room temperature.

We examine whether molecular oxygen exists in the reactor to induce overoxidation of CH_4 to CO_2. Our control experiment shows that adding gaseous oxygen into the reactor results in producing a large amount of CO_2 as well as forming H_2O instead of H_2 (Supplementary Fig. 14), consistent with the previous reports for photocatalytic oxidative coupling of methane[4]. This demonstrates that the oxidant for formation of CO_2 during NOCM is provided by the lattice oxygen of catalyst.

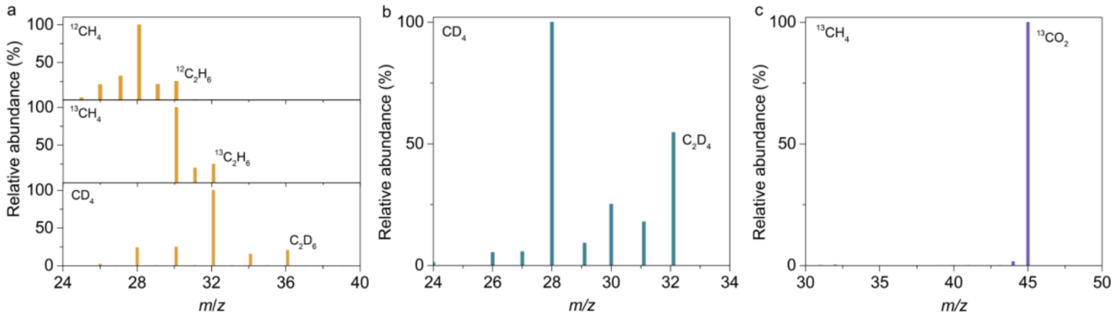

Supplementary Fig. 15 Isotope labelling measurements. (a) Mass spectra of C_2H_6 produced from NOCM process over Pd_1/TiO_2 using $^{12}CH_4$, $^{13}CH_4$ or CD_4 as the reactant. Mass spectra of (b) C_2D_4 and (c) $^{13}CO_2$ produced from NOCM process over Pd_1/TiO_2 using CD_4 or $^{13}CH_4$ as the reactant, respectively.

The origin of C_{2+} compounds is verified by using $^{13}CH_4$, $^{12}CH_4$ and deuterated methane (CD_4) as carbon source. The *m*/*z* values of C_2H_6 are shift to higher mass/charge ratios with constant relative intensity when CH_4 is replaced by $^{13}CH_4$ or CD_4 while the mass spectra of generated C_2H_4 and CO_2 show similar trends (Supplementary Fig. 15), confirming that C_2H_6 is transformed from CH_4.

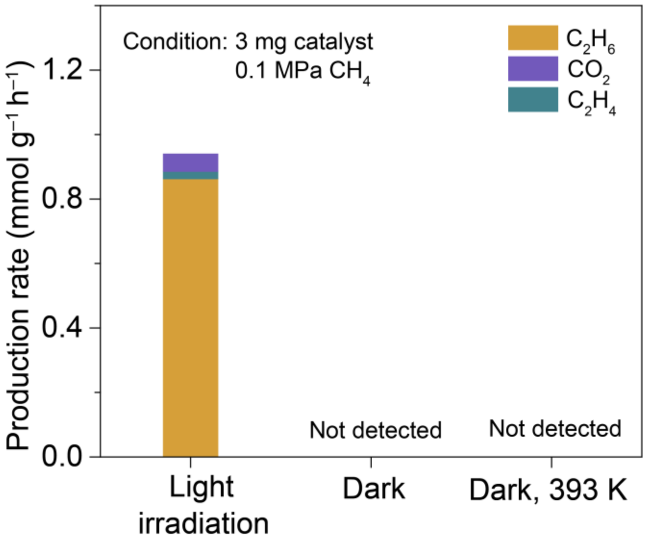

Supplementary Fig. 16 Control experiments for NOCM. The comparison of NOCM performance over Pd_1/TiO_2 in control experiments under dark condition (at room temperature or with extra 393 K heating).

Negligible carbonaceous product is formed under dark condition (Supplementary Fig. 16), suggesting that photogenerated charge carriers play the dominant role in CH_4 conversion over Pd_1/TiO_2.

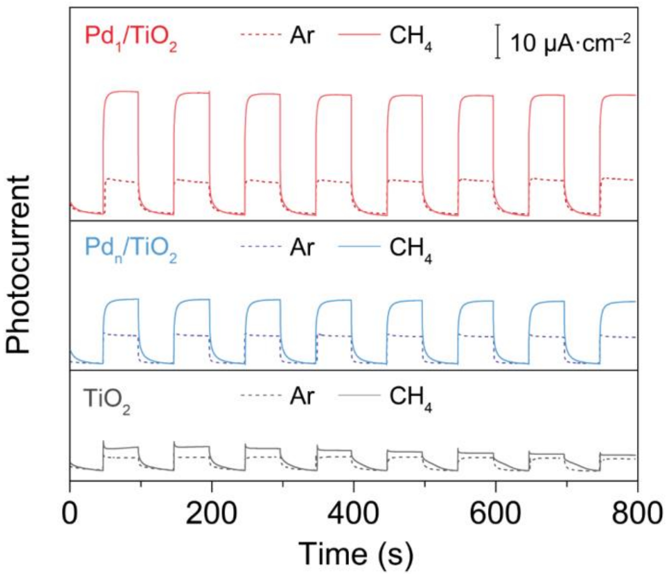

Supplementary Fig. 17 Photocurrent measurements. Photocurrent responses of as-prepared samples in CH_4 or Ar atmosphere.

To understand the utilization efficiency of photogenerated charge carriers by Pd modification, photoelectrochemical measurement is employed to examine the interfacial charge dynamics on TiO_2, Pd_n/TiO_2 and Pd_1/TiO_2 catalysts under Ar or CH_4 atmosphere. In the absence of CH_4, the photocurrents of Pd_n/TiO_2 and Pd_1/TiO_2 are larger than those of pristine TiO_2 (Supplementary Fig. 17). This indicates that Pd modification can facilitate charge separation and migration although Pd SAs and Pd nanoparticles play different roles as demonstrated by the aforementioned theoretical simulation and X-ray photoelectron spectroscopy (XPS) characterization. After introducing CH_4 atmosphere, the photocurrents are significantly enhanced in the order of $Pd_1/TiO_2 > Pd_n/TiO_2 > TiO_2$. The increase of photocurrents is attributed to the consumption of photogenerated holes by CH_4 that essentially enhances the transfer of photogenerated electrons to electrode, manifesting the better CH_4 activation on Pd_1/TiO_2[5].

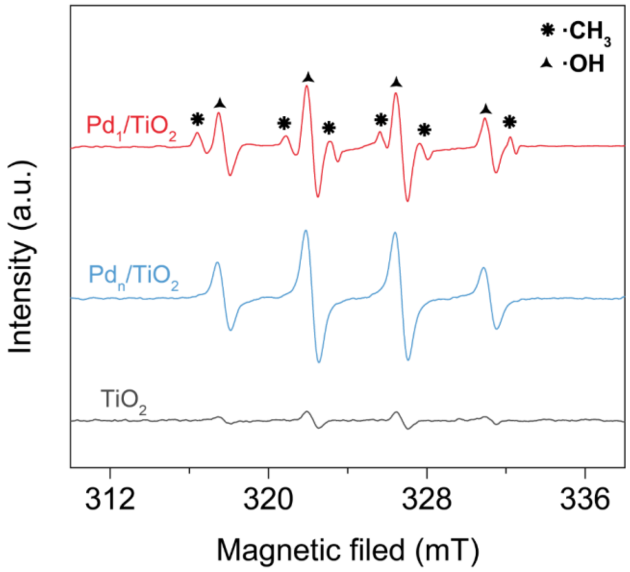

Supplementary Fig. 18 EPR characterization. EPR spectra of the samples in water with CH_4 dissolved under light irradiation.

Electron paramagnetic resonance (EPR) measurement is performed to investigate possible radicals by using 5,5′-dimethyl-1-pyrroline-N-oxide (DMPO) as a radical scavenger[6,7]. In order to detect the radicals, the reaction is required to carry out in water with CH_4 as filling gas under light irradiation.

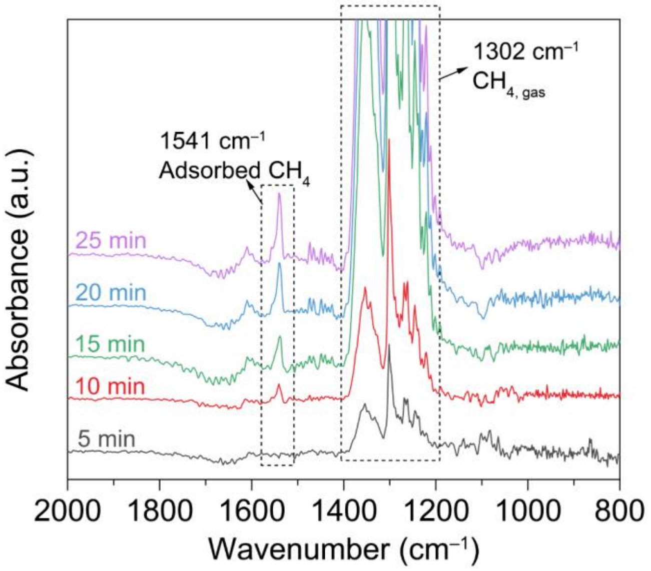

Supplementary Fig. 19 In situ DRIFTS measurements in the dark. In situ DRIFTS spectra for CH_4 adsorption on Pd_1/TiO_2 at room temperature in the dark.

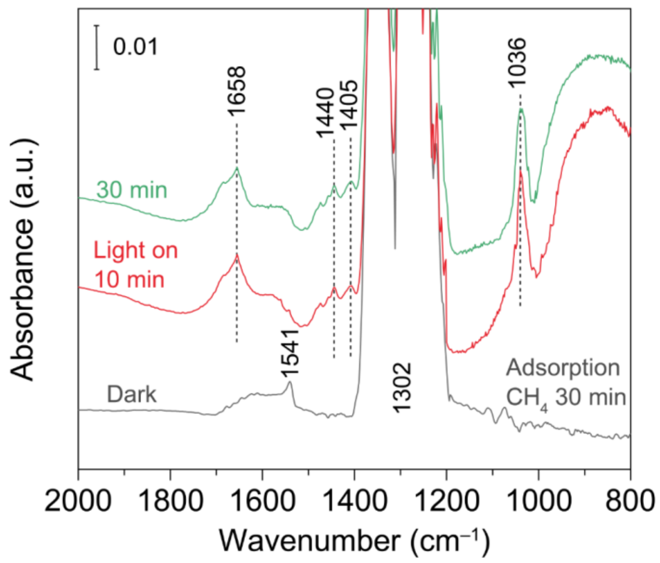

Supplementary Fig. 20 In situ DRIFTS measurements of TiO_2. In situ DRIFTS spectra for photocatalytic NOCM over pure TiO_2.

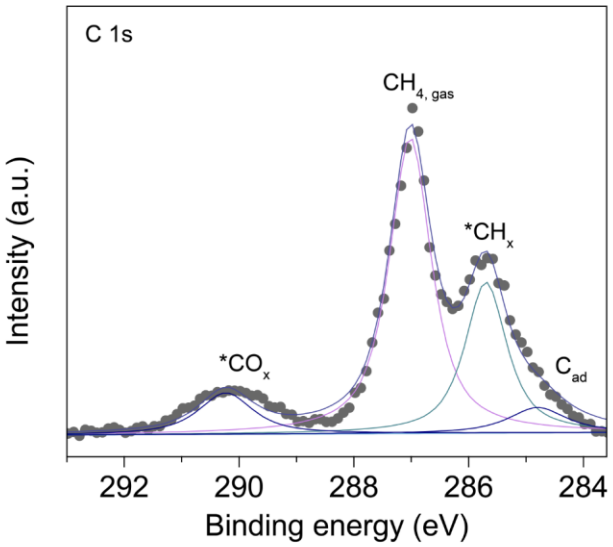

Supplementary Fig. 21 NAP-XPS measurements. C 1s near ambient pressure X-ray photoelectron spectrum (NAP-XPS) collected for CH_4 conversion over Pd_1/TiO_2 under light illumination at 60 min.

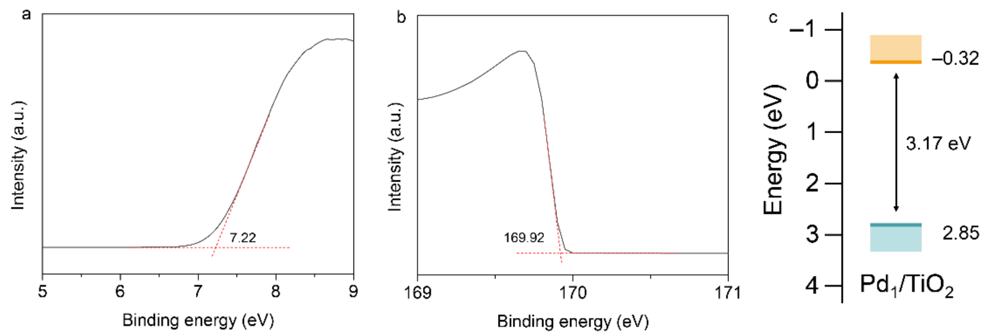

Supplementary Fig. 22 Electronic band structure. (a) Valence band spectra and (b) secondary electron cutoff for Pd_1/TiO_2. The excitation photon energy is 170 eV. (c) The electronic band structure of Pd_1/TiO_2.

The U_h is the energy level of VBM (hole potential) relative to the standard hydrogen electrode (SHE), which is obtained according to the valence band position of Pd_1/TiO_2 measured by ultraviolet photoelectron spectroscopy (UPS).

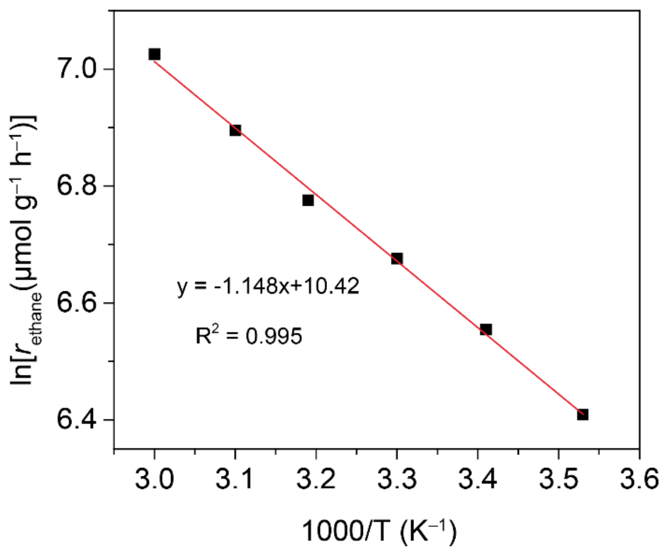

Supplementary Fig. 23 Arrhenius plot. Arrhenius plot of NOCM rate over Pd_1/TiO_2 from 283 to 333 K.

We experimentally measure the activation energy of the NOCM reaction according to the Arrhenius equation, $c = Ae^{-Ea/RT}$, in which *c* is ethane production rate, *A* is Arrhenius constants, *Ea* is apparent activation energy, *R* is gas constant and *T* is reaction temperature.

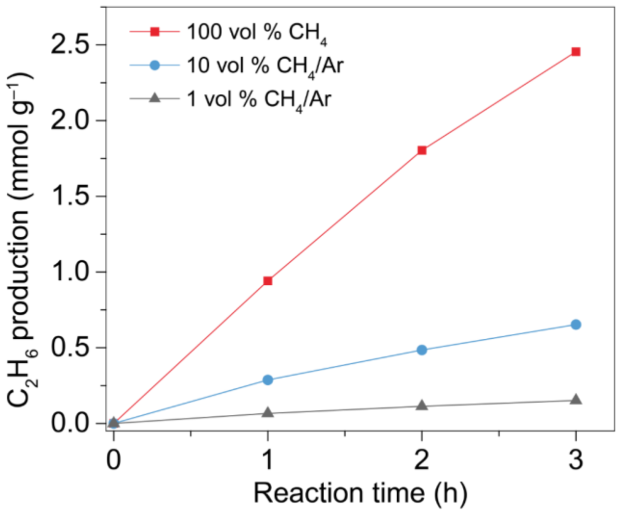

Supplementary Fig. 24 NOCM performance with different CH_4 concentrations. The production of C_2H_6 in photocatalytic NOCM over Pd_1/TiO_2 in 0.1 MPa pure CH_4, 10 vol.% CH_4/Ar or 1 vol.% CH_4/Ar.

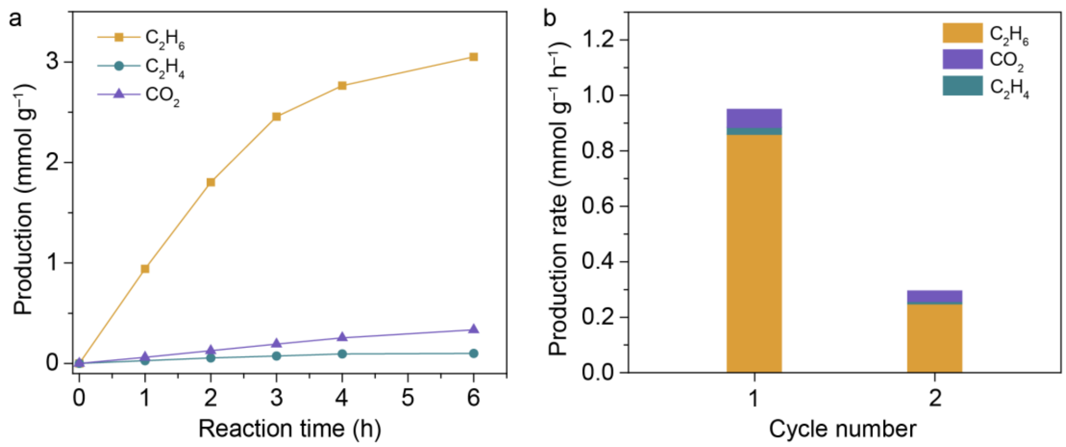

Supplementary Fig. 25 Performance stability. (a) The time-dependent production of carbonaceous products in photocatalytic NOCM over Pd_1/TiO_2. (b) Production rates of carbonaceous products in the cyclic tests by Pd_1/TiO_2 without regeneration treatment. Each cycle lasts 3 h.

The time-dependent NOCM on Pd_1/TiO_2 shows that the performance decays when the reaction time reaches 6 h (Supplementary Fig. 25a), which should be ascribed to the consumption of lattice oxygen in TiO_2 for CO_2 production. The catalytic performance for the first and second cycles is displayed in Supplementary Fig. 25b. Without catalyst regeneration treatment, the yields of C_2H_6 and CO_2 in the second cycle are 3.5 and 2.1 times lower than that in the first cycle, respectively.

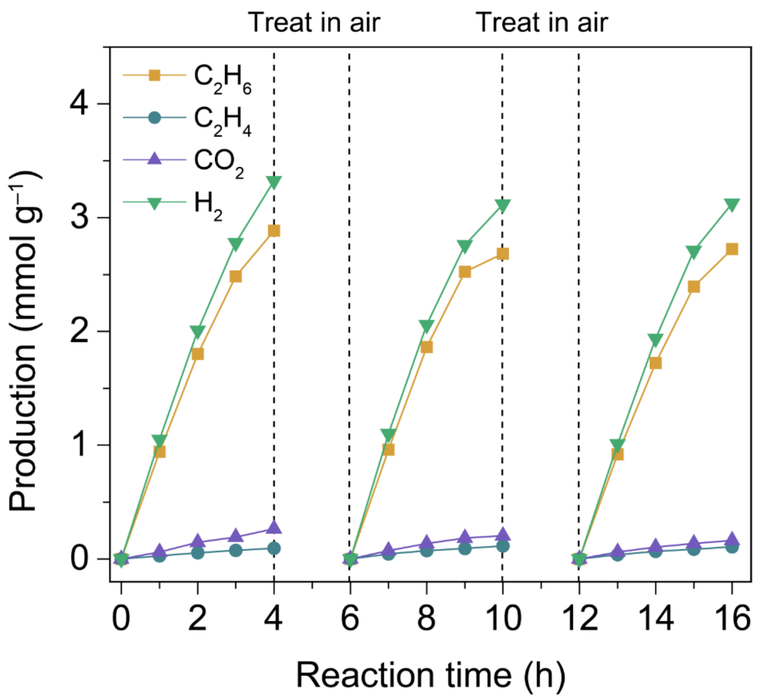

Supplementary Fig. 26 Catalyst recycling. Reaction–regeneration cycles in photocatalytic NOCM over Pd_1/TiO_2. The regeneration treatment exposes catalyst to air with 80 ℃ heating.

The consumed lattice oxygen can be regenerated by heating the catalyst in air, recovering the photocatalytic activity. As shown in Supplementary Fig. 26, the yields of carbonaceous products and H_2 are recovered after the heating treatment over Pd_1/TiO_2 in the air.

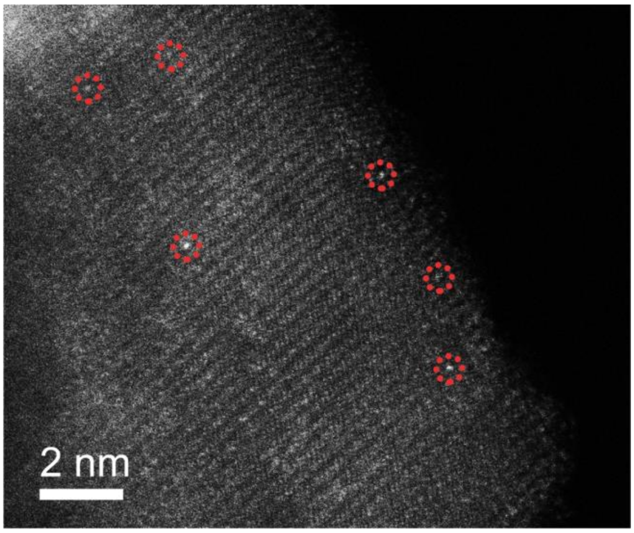

Supplementary Fig. 27 Catalyst characterization after NOCM. High-angle annular dark-field scanning transmission electron microscopy (HAADF-STEM) image of Pd_1/TiO_2 catalyst after photocatalytic NOCM measurements.

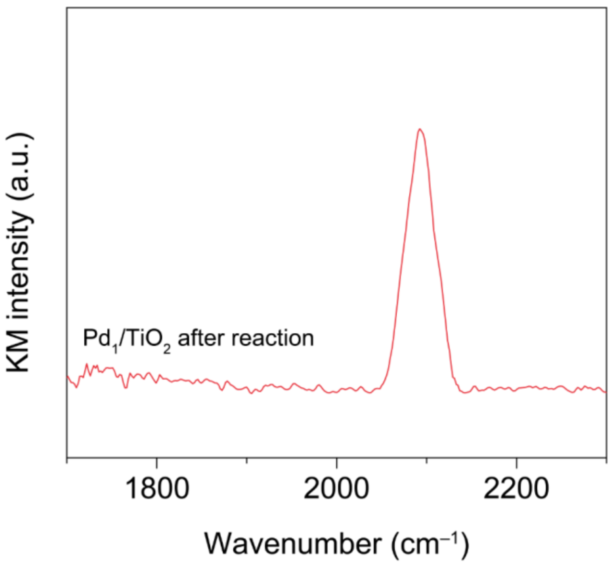

Supplementary Fig. 28 CO adsorption DRIFTS characterization after NOCM. CO adsorption DRIFTS spectrum of Pd_1/TiO_2 after photocatalytic NOCM measurements.

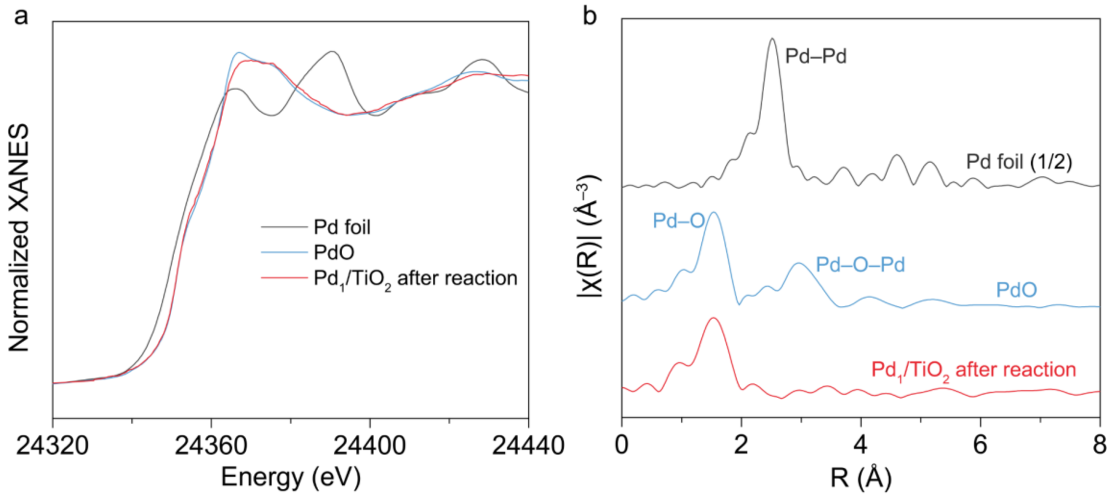

Supplementary Fig. 29 XANES and EXAFS characterization after NOCM. (a) Normalized Pd K-edge X-ray absorption near-edge structure (XANES) spectra and (b) extended X-ray adsorption fine structure (EXAFS) spectra in *R* space of Pd_1/TiO_2 after photocatalytic NOCM measurements, in reference to Pd foil and PdO.

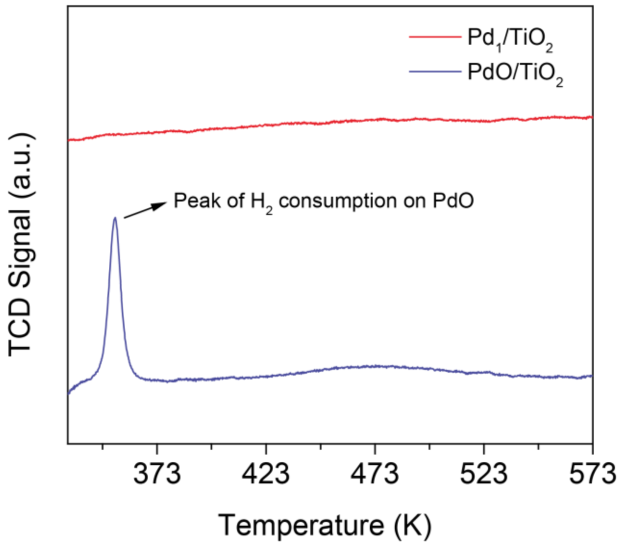

Supplementary Fig. 30 H_2–TPR analysis of Pd_1/TiO_2 and PdO/TiO_2. The PdO/TiO_2 is obtained by calcinating Pd_n/TiO_2 at 450 °C for 2 h.

The H_2–temperature programmed reduction (TPR) profile of PdO/TiO_2 catalyst exhibits one peak of H_2 consumption at 353 K, representing the reduction of PdO species on TiO_2[8]. In contrast, no signal for H_2 consumption can be observed for Pd_1/TiO_2 during H_2–TPR measurement. This indicates that the Pd–O species in Pd_1/TiO_2 are more stable than Pd oxide, which enables high activity and stability for photocatalytic NOCM.

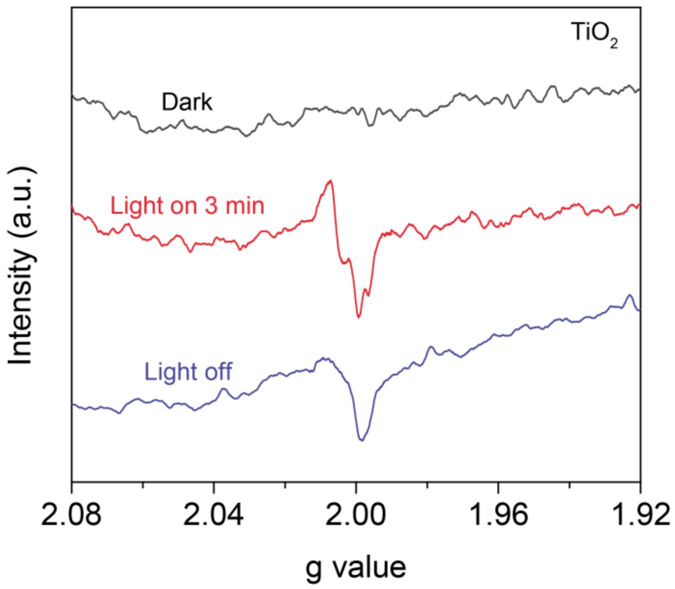

Supplementary Fig. 31 In situ EPR characterization. In situ EPR spectra for TiO_2 catalyst in 0.1 MPa CH_4 under different conditions.

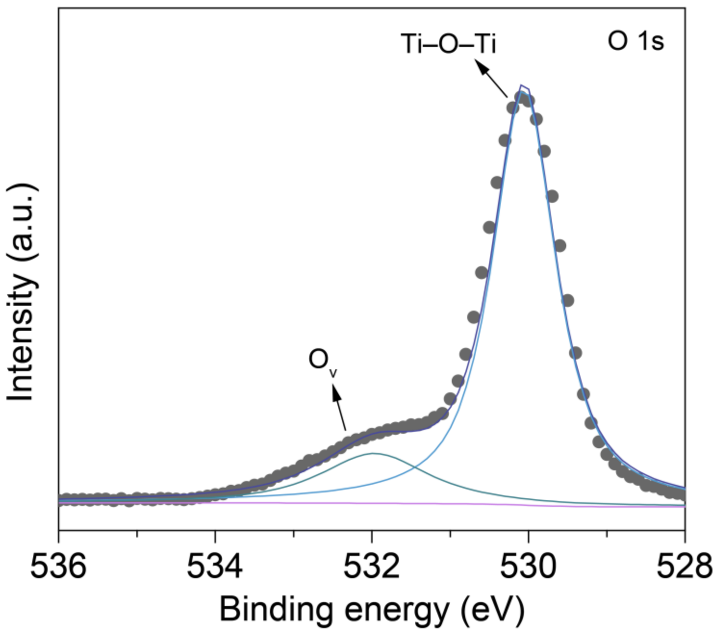

Supplementary Fig. 32 In situ XPS characterization. In situ high-resolution O 1s XPS spectra and peak fittings for Pd_1/TiO_2 catalyst after light illumination in 45 Pa of CH_4.

Apart from the peak at 530 eV deemed to the Ti–O–Ti bond, the O 1s peak at 531.8 eV ascribed to the O atom near the O vacancy gradually appears (Supplementary Fig. 32)[9,10]. Such a peak associated with O vacancy is reserved when the light is turned off. The increased content of O vacancies, observed on the surface of Pd_1/TiO_2 during the reaction, further demonstrates that lattice oxygen is consumed during photocatalysis to form CO_2 by following the pathway in Supplementary Fig. 33.

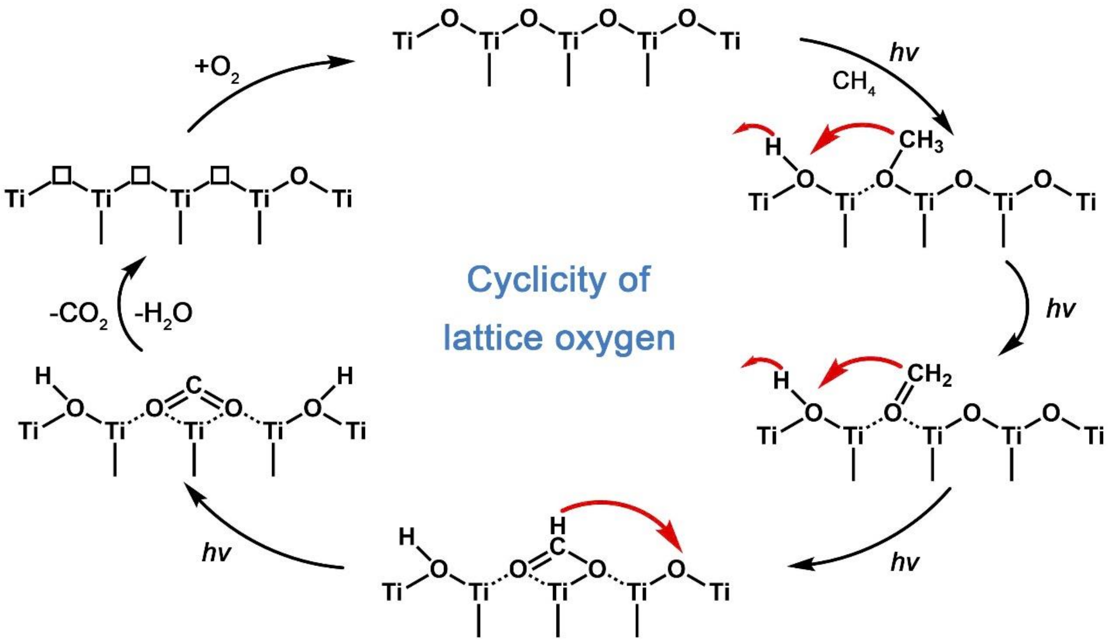

Supplementary Fig. 33 Proposed mechanism. Proposed reaction pathway for forming CO_2 and O vacancies during photocatalytic NOCM over Pd_1/TiO_2 catalyst.

For pure TiO_2 catalyst, CH_4 is dissociated at the O sites of TiO_2 that accumulate photogenerated holes. Most of the activated CH_4 would be further oxidized to produce CO_2, creating O vacancies (Supplementary Fig. 33). Notably, only a small amount of H_2 can be detected in our measurement, implying that a large proportion of H atoms are combined with lattice oxygen to form OH or H_2O species.

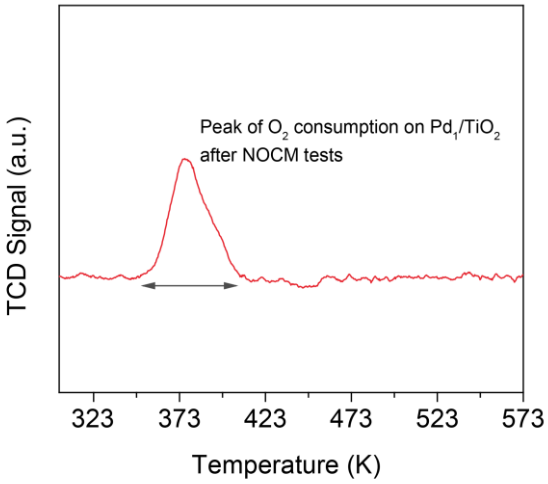

Supplementary Fig. 34 TPO characterization after NOCM. TPO of Pd_1/TiO_2 catalyst after photocatalytic NOCM tests, using 1% O_2/He. Reaction condition: 60 mg catalyst, 0.1 MPa CH_4, 4 h light irradiation.

As shown in Supplementary Fig. 34, when the temperature is increased during temperature-programmed oxidation (TPO), the peak starting around 348 K indicates that sample oxidation can occur at low temperature by O_2. Furthermore, the change of O content (N_O) can be determined using the relationship $N_O = 32 \times C_O/m_{TPO}$, where C_O is the O_2 consumption measured by TPO and m_{TPO} is the mass of TPO sample with 32 being the molar mass of O_2. The C_O is 13.2 μmol measured by TPO, and as such, the N_O is determined to be 0.7 wt.%.

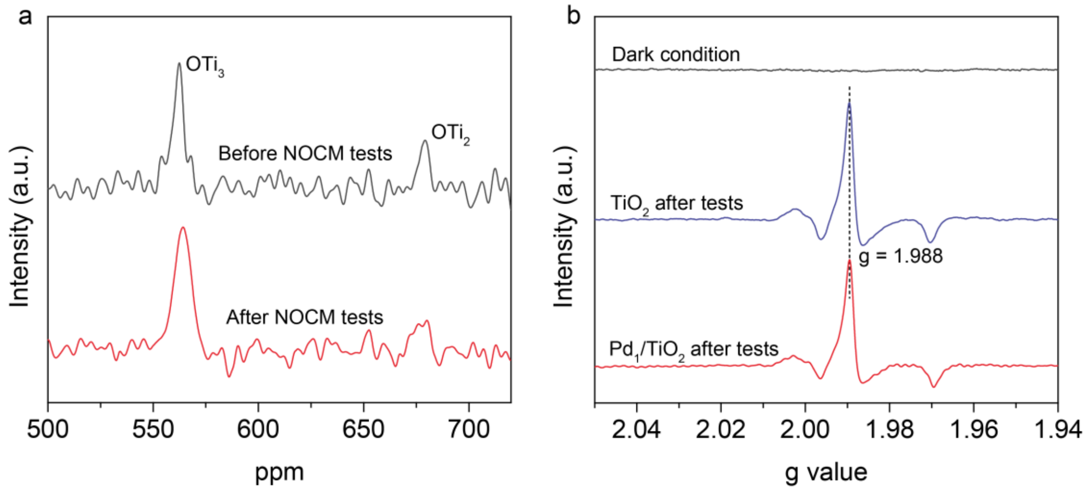

Supplementary Fig. 35 NMR and EPR characterization after NOCM. (a) ^{17}O MAS NMR spectra of Pd_1/TiO_2 before and after photocatalytic NOCM measurements. (b) EPR spectra of Pd_1/TiO_2 and TiO_2 after photocatalytic NOCM measurements under 77 K.

The ^{17}O magic angle spinning nuclear magnetic resonance (^{17}O MAS NMR) spectra of Pd_1/TiO_2 before and after photocatalytic NOCM measurements are shown in Supplementary Fig. 35a. The peaks at 562 and 678 ppm correspond to the ^{17}O resonance of OTi_3 and OTi_2 [11]. The peak intensity at 678 ppm decreases after NOCM measurements, indicating that the content of oxygen with 2-fold (OTi_2) oxide coordination on TiO_2 surface has been reduced. However, the signal-to-noise ratio of ^{17}O MAS NMR spectra is unsatisfactory as the abundance of ^{17}O is extremely low in the nature. Moreover, the long collection time for ^{17}O MAS NMR spectra (more than 20 h) may lead to the unexpected oxidation of catalyst by the air. For this reason, the EPR is employed to further verify the location of O consumption during the NOCM process. As shown in Supplementary Fig. 35b, both the TiO_2 and Pd_1/TiO_2 show the same peak at g = 1.988, indicating the appearance of Ti^{3+} after the NOCM measurements[12]. Moreover, the intensity of Ti^{3+} is similar in TiO_2 and Pd_1/TiO_2, suggesting that the consumption of lattice oxygen takes place near the Ti sites rather than Pd sites.

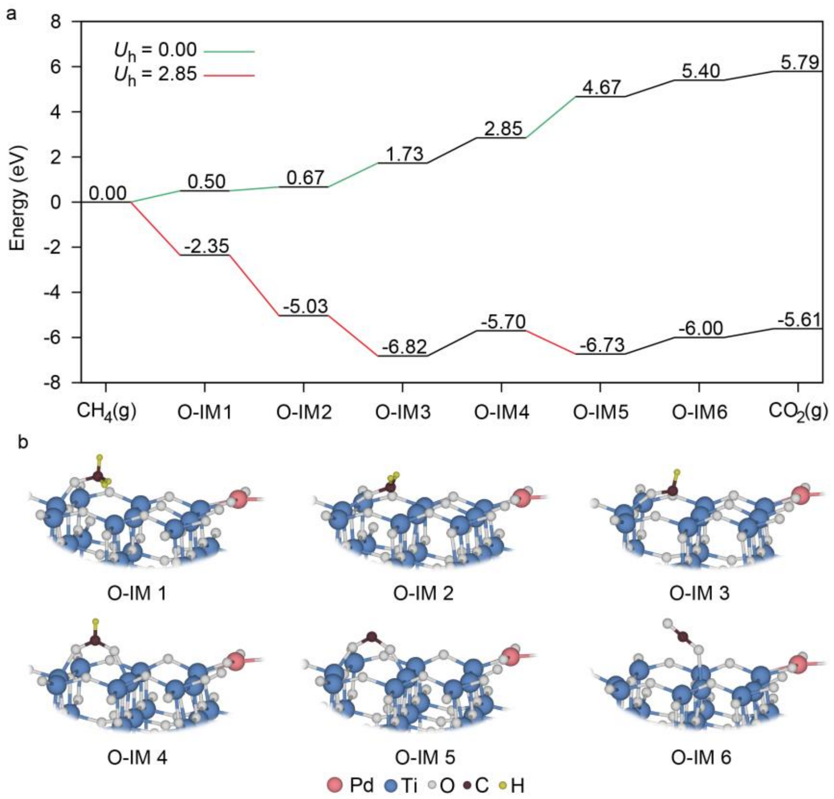

Supplementary Fig. 36 Calculation for CH_4 overoxidation on Pd_1/TiO_2. (a) Free-energy diagrams for overoxidation of CH_4 on Pd_1/TiO_2 with the hole energy of 2.85 eV. (b) Atomic configurations for the corresponding steps in the simulation.

As shown in Supplementary Fig. 36, the photogenerated holes are required to provide extra potential for CH_4 dehydrogenation on O site; otherwise, the high energy barrier of these steps will inhibit the process thermodynamically (i.e., green and red lines in the energy diagrams). Specifically, the activation of the last hydrogen atom in CH_4 requires a high energy of 1.82 eV (i.e., green line between O-IM4 and O-IM5), which is the rate-determining step for the overoxidation process under dark condition. However, the energy barrier of this step turns into downhill with the assistance of photogenerated holes (i.e., red line between O-IM4 and O-IM5), and the bonding between the adsorbed CH_4 and the second lattice oxygen becomes the rate-determining step with the energy barrier of 1.12 eV (i.e., O-IM3 to O-IM4), indicating that the stabilization of the second lattice oxygen can weaken the bonding between CH_4 and O site and suppress the overoxidation of CH_4

under light irradiation.

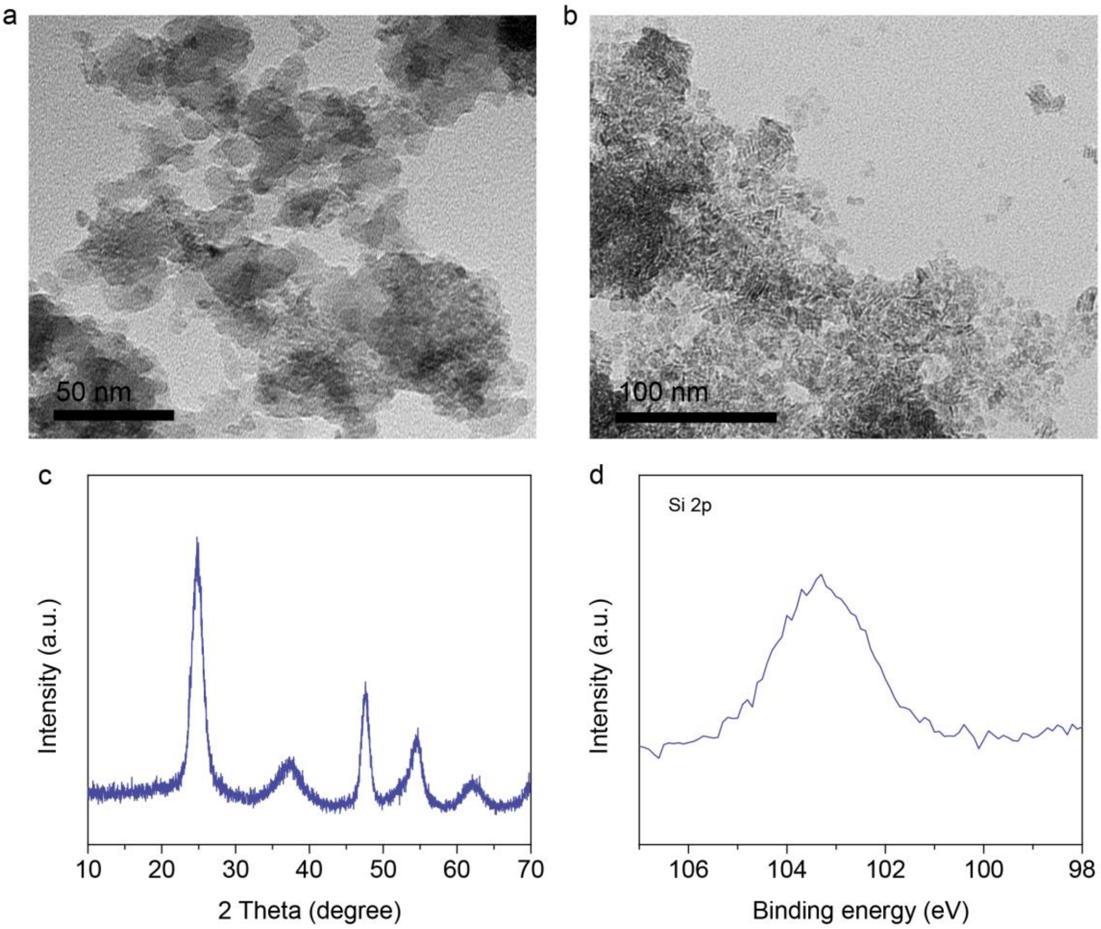

Supplementary Fig. 37 Characterization for Pd_1/ST. (a, b) TEM images, (c) XRD pattern and (d) high-resolution Si 2p XPS spectrum of Pd_1/ST.

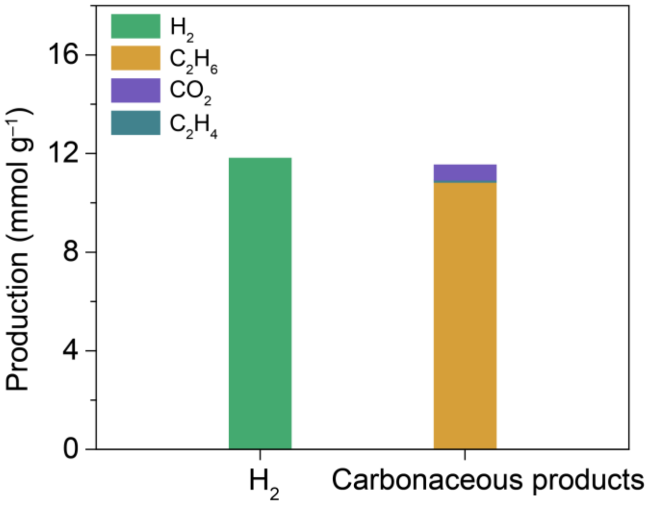

Supplementary Fig. 38 NOCM performance for Pd_1/ST. The production of carbonaceous products and H_2 in photocatalytic NOCM over Pd_1/ST after 24 h light irradiation.

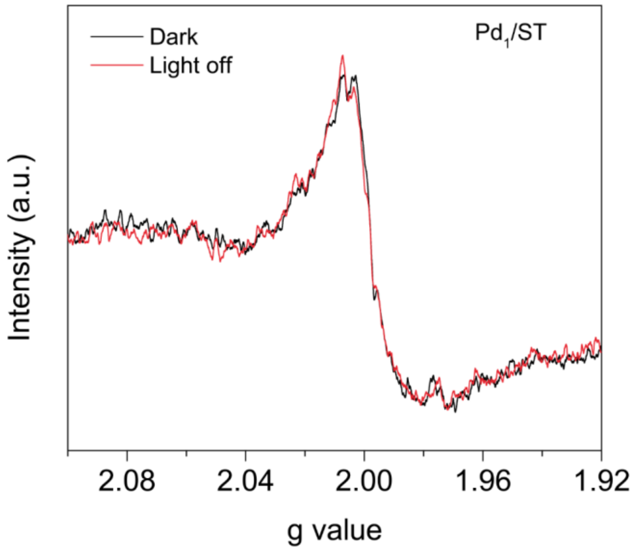

Supplementary Fig. 39 In situ EPR measurement for Pd_1/ST. In situ EPR spectra for Pd_1/ST catalyst in 0.1 MPa CH_4 before and after light irradiation.

According to the work by Saputera et al.[13], the incorporation of Si into TiO_2 lattice can generate Ti atoms with lower valence state, resulting in the signal of O defects in EPR spectrum. Nevertheless, the EPR signal intensity for O defects in Pd_1/ST is almost maintained after photocatalytic NOCM. This indicates that the photocatalytic NOCM over Pd_1/ST does not create more O vacancies, demonstrating that Si doping can stabilize lattice oxygen.

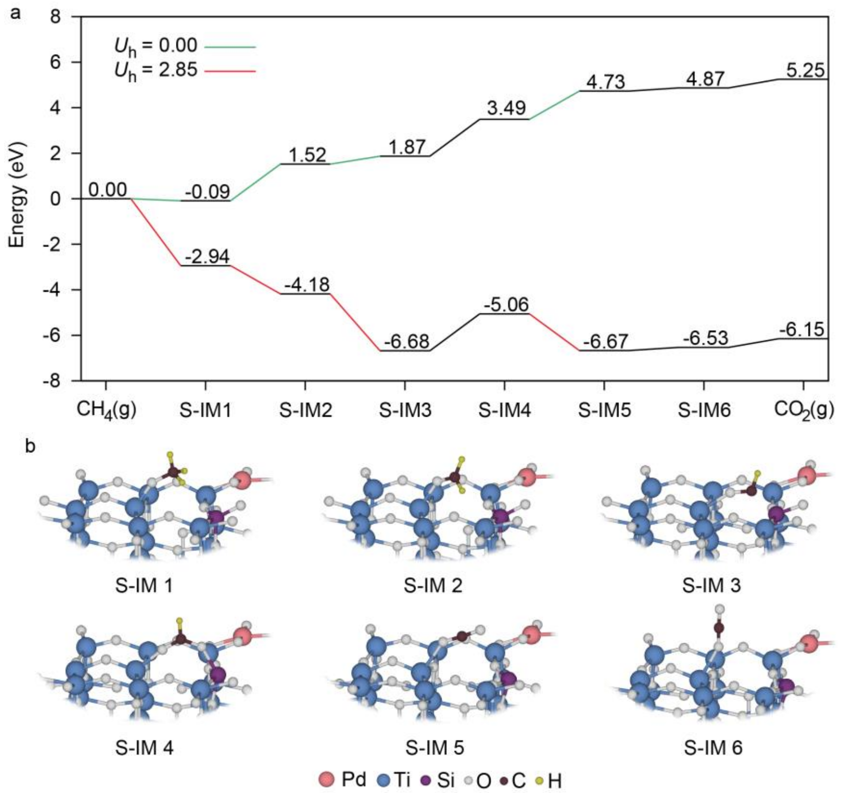

Supplementary Fig. 40 Calculation for CH_4 overoxidation on Pd_1/ST. (a) Free-energy diagrams for overoxidation of CH_4 on Pd_1/ST with the hole energy of 2.85 eV. (b) Atomic configurations for the corresponding steps in the simulation.

The reaction steps and the corresponding atomic configurations over Pd_1/ST are similar to those of Pd_1/TiO_2, and the step of S-IM3 to S-IM4 is still the key step for CH_4 overoxidation under light irradiation. However, this bonding step between the adsorbed CH_4 and the second lattice oxygen displays the energy barrier of 1.62 eV on Pd_1/ST, dramatically higher than that on Pd_1/TiO_2 (1.12 eV), indicating that the incorporation of Si atoms into TiO_2 can suppress the overoxidation of CH_4 by stabilizing lattice oxygen.

Supplementary Table 1. **Pd content of catalysts.** The usage of $Pd(NO_3)_2$ solution (21.1 mM) in the synthesis of Pd-modified TiO_2 samples, and the corresponding contents of Pd in the final samples. The content of Pd in the samples is determined by inductively coupled plasma–mass spectrometry (ICP–MS).

Sample	Usage of $Pd(NO_3)_2$	Content of Pd
Pd_1/TiO_2	20 μL	0.2 wt.%
Pd_n/TiO_2	20 μL	0.23 wt.%

Supplementary Table 2. **EXAFS fitting.** Fitting results of Pd K-Edge EXAFS data.[a]

Sample	Path	CN	R (Å)	σ^2 (Å)
Pd foil	Pd–Pd	12 (fixed)	2.74 ± 0.01	0.00570
Pd_1/TiO_2	Pd–O	4.53 ± 0.57	2.02 ± 0.01	0.00343

[a] CN, the coordination number; R, the bond length; σ^2, the Debye-Waller factor

Supplementary Table 3. **Carbon balance (CB).** The CB for photocatalytic NOCM process over Pd_1/TiO_2 catalyst.

Reaction time	Consumption	Production				CB
	CH_4	C_2H_6	C_2H_4	CO_2	CO	
1 h	6.17 μmol	2.76 μmol	0.087 μmol	0.171 μmol	N.D.	95.1%
3 h	16.98 μmol	7.55 μmol	0.198 μmol	0.516 μmol	N.D.	94.3%

Supplementary Table 4. **Performance comparison.** Representative works on photocatalytic NOCM reaction.

Catalyst	Reaction condition	C_2H_6 production	Apparent quantum efficiency	Stability in one cycle	Reference
Pd_1/TiO_2	3.0 mg catalyst; 1338 μmol CH_4	913 μmol g^{-1} h^{-1}	3.05% at 350 nm	6 h	This work
Pd_1/TiO_2	3.0 mg catalyst; 133.8 μmol CH_4 (10 vol%)	287 μmol g^{-1} h^{-1}	0.96% at 350 nm	5-6 h	This work
Pd_1/TiO_2	3.0 mg catalyst; 13.38 μmol CH_4 (1 vol%)	61 μmol g^{-1} h^{-1}	0.203% at 350 nm	5-6 h	This work
Pd_1/ST	3.0 mg catalyst; 1338 μmol CH_4	802 μmol g^{-1} h^{-1}	2.41% at 350 nm	24 h	This work
(Zn^{+},Zn^{2+})-ZSM-5^{-}	1.0 g catalyst; 200 μmol CH_4	4.9 μmol g^{-1} h^{-1}	0.55% at 300-400 nm	Not report (NR)	14
Ga-ETS-10-0.2	0.2 g catalyst; 200 μmol CH_4	11.86 μmol g^{-1} h^{-1}	NR	5 h	15
Au/m-ZnO-4.8	1.0 mg catalyst; 22.3 μmol CH_4	11.42 μmol g^{-1} h^{-1}	NR	12 h (only CH_4)	16
Pt/HGTS (2%)	0.2 g catalyst; 44.6 μmol CH_4	1.68 μmol g^{-1} h^{-1}	0.01% at 350 nm	12 h	17
Ag-HPW/TiO_2	0.1 g catalyst; 0.3 MPa CH_4	22.07 μmol g^{-1} h^{-1}	3.5% at 362 nm	7 h	18
2Nb-TS	0.1 g catalyst; 44.6 μmol CH_4	1.69 μmol g^{-1} h^{-1}	NR	16 h	19
Au/TiO_2	5.0 mg catalyst; 10 vol% CH_4 flow rate	81.7 μmol g^{-1} h^{-1}	NR	NR	20
SS-F	2.0 mg catalyst; 300 μmol CH_4	163 μmol g^{-1} h^{-1}	0.72% at 325 nm	NR	21

Supplementary References

1. Pennington, A. M. et al. Photocatalytic CO oxidation over nanoparticulate Au-modified TiO_2 aerogels: the importance of size and intimacy. *ACS Catal.* **10,** 14834-14846 (2020).
2. Liu, P. et al. Photochemical route for synthesizing atomically dispersed palladium catalysts. *Science* **352,** 797 (2016).
3. Zhang, Y. et al. Structure sensitivity of Au-TiO_2 strong metal–support interactions. *Angew. Chem. Int. Ed.* **60,** 12074-12081 (2021).
4. Li, X. et al. Platinum- and CuO_x-decorated TiO_2 photocatalyst for oxidative coupling of methane to C_2 hydrocarbons in a flow reactor. *Angew. Chem. Int. Ed.* **59,** 19702-19707 (2020).
5. Li, H., Shang, J., Ai, Z. & Zhang, L. Efficient visible light nitrogen fixation with BiOBr nanosheets of oxygen vacancies on the exposed {001} facets. *J. Am. Chem. Soc.* **137,** 6393-6399 (2015).
6. Bai, S. et al. High-efficiency direct methane conversion to oxygenates on a cerium dioxide nanowires supported rhodium single-atom catalyst. *Nat. Commun.* **11,** 954 (2020).
7. Shen, Q. et al. Single chromium atoms supported on titanium dioxide nanoparticles for synergic catalytic methane conversion under mild conditions. *Angew. Chem. Int. Ed.* **59,** 1216-1219 (2020).
8. Zhou, R., Zhao, B. & Yue, B. Effects of CeO_2-ZrO_2 present in Pd/Al_2O_3 catalysts on the redox behavior of PdO_x and their combustion activity. *Appl. Surf. Sci.* **254,** 4701-4707 (2008).
9. Wan, J. et al. Defect effects on TiO_2 nanosheets: stabilizing single atomic site Au and promoting catalytic properties. *Adv. Mater.* **30,** 1705369 (2018).
10. Ou, G. et al. Tuning defects in oxides at room temperature by lithium reduction. *Nat. Commun.* **9,** 1302 (2018).
11. Rao, Y. et al. ^{17}O and ^{15}N solid state NMR studies on ligand-assisted templating and oxygen coordination in the walls of mesoporous Nb, Ta and Ti oxides. *J. Am. Chem. Soc.* **130,** 15726-15731 (2008).
12. Liu, Y. et al. Engineering self-doped surface defects of anatase TiO_2 nanosheets for enhanced photocatalytic efficiency. *Appl. Surf. Sci.* **540,** 148330 (2021).
13. Saputera, W. H. et al. Light-induced synergistic multidefect sites on TiO_2/SiO_2 composites for catalytic dehydrogenation. *ACS Catal.* **9,** 2674-2684 (2019).
14. Li, L. et al. Efficient sunlight-driven dehydrogenative coupling of methane to ethane over a Zn^{+}-modified zeolite. *Angew. Chem. Int. Ed.* **50,** 8299-8303 (2011).
15. Li, L. et al. Synergistic effect on the photoactivation of the methane C–H bond over Ga^{3+}-modified ETS-10. *Angew. Chem. Int. Ed.* **51,** 4702-4706 (2012).
16. Meng, L. et al. Gold plasmon-induced photocatalytic dehydrogenative coupling of methane to ethane on polar oxide surfaces. *Energy Environ. Sci.* **11,** 294-298 (2018).
17. Wu, S. et al. Ga-doped and Pt-loaded porous TiO_2–SiO_2 for photocatalytic nonoxidative coupling of methane. *J. Am. Chem. Soc.* **141,** 6592-6600 (2019).
18. Yu, X. et al. Stoichiometric methane conversion to ethane using photochemical looping at ambient temperature. *Nat. Energy* **5,** 511-519 (2020).
19. Chen, Z. et al. Non-oxidative coupling of methane: N-type doping of niobium single atoms in TiO_2–SiO_2 induces electron localization. *Angew. Chem. Int. Ed.* **60,** 11901-11909 (2021).
20. Lang, J. et al. Highly efficient light-driven methane coupling under ambient conditions based on an integrated design of a photocatalytic system. *Green Chem.* **22,** 4669-4675 (2020).
21. Wang, G. et al. Light-induced nonoxidative coupling of methane using stable solid solutions. *Angew. Chem. Int. Ed.* **60,** 20760-20764 (2021).
